# Leucopterin, the white pigment in butterfly wings: structural analysis by PDF fit, FIDEL fit, Rietveld refinement, solid-state NMR and DFT-D

**DOI:** 10.1107/S2052252523004281

**Published:** 2023-06-17

**Authors:** Federica Bravetti, Lukas Tapmeyer, Kathrin Skorodumov, Edith Alig, Stefan Habermehl, Robert Hühn, Simone Bordignon, Angelo Gallo, Carlo Nervi, Michele R. Chierotti, Martin U. Schmidt

**Affiliations:** a Dipartimento di Chimica e Centro di Eccellenza NIS, Università degli Studi di Torino, Via Pietro Giuria 7, 10125 Torino, Italy; b Institut für Anorganische und Analytische Chemie, Goethe-Universität, Max-von-Laue-Strasse 7, 60438 Frankfurt am Main, Germany; c F.K.M. Buster Altöl- und Reststoff-Entsorgung GmbH, Holländerstrasse 18, 68219 Mannheim, Germany; d Institut für Organische Chemie und Chemische Biologie, Goethe-Universität, Max-von-Laue-Strasse 7, 60438 Frankfurt am Main, Germany; Formby, Liverpool, United Kingdom

**Keywords:** structure determination, powder data, pair distribution function, PDF, Rietveld refinement, solid-state NMR, DFT-D, chemical shift calculation, leucopterin, organic white pigment, nonstoichiometric hydrate, FIDEL, high density

## Abstract

Leucopterin (C_6_H_5_N_5_O_3_) is the white pigment in the wings of *Pieris brassicae* butterflies, and other butterflies; it can also be found in wasps and other insects. Its crystal structure and its tautomeric form in the solid state were hitherto unknown. Leucopterin turned out to be a variable hydrate, with 0.5 to about 0.1 molecules of water per leucopterin molecule. Under ambient conditions, the preferred state is the hemihydrate. Initially, all attempts to grow single crystals suitable for X-ray diffraction were to no avail. Attempts to determine the crystal structure by powder diffraction using the direct-space method failed, because the trials did not include the correct, but rare, space group *P*2/*c*. Attempts were made to solve the crystal structure by a global fit to the pair distribution function (PDF-Global-Fit), as described by Prill and co-workers [Schlesinger *et al.* (2021 ▸). *J. Appl. Cryst.* **54**, 776–786]. The approach worked well, but the correct structure was not found, because again the correct space group was not included. Finally, tiny single crystals of the hemihydrate could be obtained, which allowed at least the determination of the crystal symmetry and the positions of the C, N and O atoms. The tautomeric state of the hemihydrate was assessed by multinuclear solid-state NMR spectroscopy. ^15^N CPMAS spectra showed the presence of one NH_2_ and three NH groups, and one unprotonated N atom, which agreed with the ^1^H MAS and ^13^C CPMAS spectra. Independently, the tautomeric state was investigated by lattice-energy minimizations with dispersion-corrected density functional theory (DFT-D) on 17 different possible tautomers, which also included the prediction of the corresponding ^1^H, ^13^C and ^15^N chemical shifts in the solid. All methods showed the presence of the 2-amino-3,5,8-H tautomer. The DFT-D calculations also confirmed the crystal structure. Heating of the hemihydrate results in a slow release of water between 130 and 250 °C, as shown by differential thermal analysis and thermogravimetry (DTA-TG). Temperature-dependent powder X-ray diffraction (PXRD) showed an irre­versible continuous shift of the reflections upon heating, which reveals that leu­cop­terin is a variable hydrate. This observation was also confirmed by PXRD of samples obtained under various synthetic and drying conditions. The crystal structure of a sample with about 0.2 molecules of water per leucopterin was solved by a fit with deviating lattice parameters (FIDEL), as described by Habermehl *et al.* [*Acta Cryst.* (2022 ▸), B**78**, 195–213]. A local fit, starting from the structure of the hemihydrate, as well as a global fit, starting from random structures, were per­formed, followed by Rietveld refinements. Despite dehydration, the space group remains *P*2/*c*. In both structures (hemihydrate and variable hydrate), the leu­cop­terin molecules are connected by 2–4 hydrogen bonds into chains, which are connected by further hydrogen bonds to neighbouring chains. The molecular packing is very efficient. The density of leucopterin hemihydrate is as high as 1.909 kg dm^−3^, which is one of the highest densities for organic compounds con­sisting of C, H, N and O only. The high density might explain the good light-scattering and opacity properties of the wings of *Pieris brassicae* and other butterflies.

## Abstract 2

Leucopterin, the white pigment in butterfly wings, was found to be a non-stoichiometric hydrate with an extremely high density. The crystal structure and the tautomeric state were investigated by single-crystal and powder XRD, solid-state NMR, DFT-D, Fit with Deviating Lattice parameters (FIDEL) and PDF global fit.

## 1. Introduction

**Figure 1:**
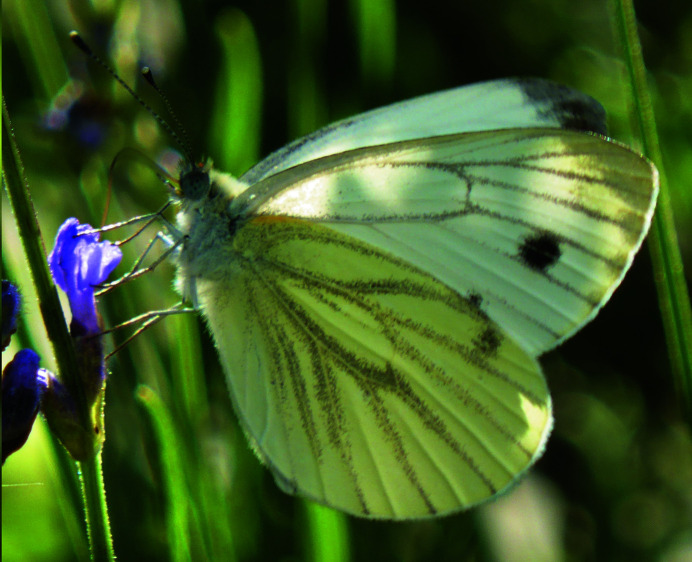
*Pieris napi* (Photo: Robert Hühn, Frankfurt am Main, 23 June 2022).

Leucopterin (2-amino-5,8-di­hydro-3*H*-pteridine-4,6,7-trione; see Scheme 1) is the white pigment in the wings of butterflies (Lepidoptera) belonging to the family Pieridae (in German ‘Weißlinge’), *e.g. Pieris brassicae* (large white or large cabbage butterfly, in German ‘Großer Kohlweißling’) or *Pieris napi* (green-veined white, in German ‘Rapsweißling’, Fig. 1 ▸).^1^ Also the name ‘leucopterin’ originates from ‘leukos’ (λɛυκóσ) = white and ‘pteron’ (πτɛρóν) = wing.^1^ 
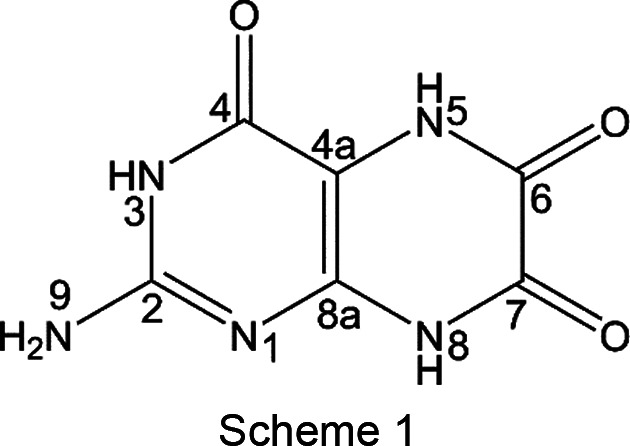


In 1926, leucopterin was isolated from about 25 000 individuals of *Pieris napi*, yielding 5.6 g of leucopterin. In 1928–1930, Bavarian school children collected about 200 000 individuals of white Pieridae butterflies under the supervision of their teachers and initiated by the Bavarian ministry of education. Additionally, about 16 000 individuals were col­lected in the region of Freiburg/Germany (Wieland *et al.*, 1933 ▸). From the 216 000 butterflies, 39.1 g of raw leucopterin were extracted (Wieland *et al.*, 1933 ▸). Despite extensive chemical investigation, the molecular structure of leucopterin remained unclear (Wieland & Purrmann, 1940 ▸). Finally, Purrmann produced leucopterin according to Scheme 2. This synthetic leucopterin agreed in all properties with natural leucopterin from butterfly wings, even in its Debye–Scherrer diagrams; see Fig. S1 in the supporting information (Purrmann, 1940 ▸). 
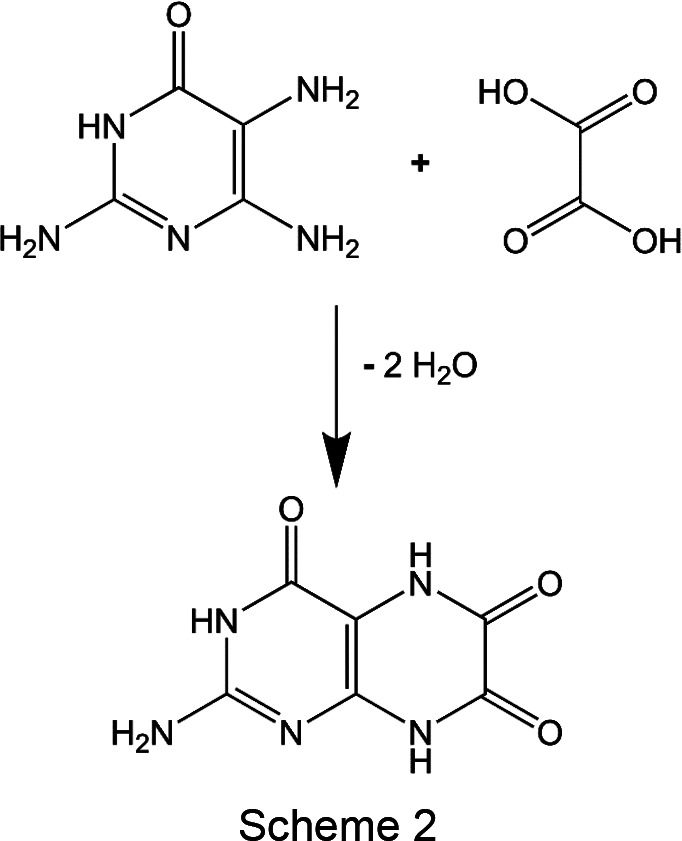


In nature, as well as in industry, organic white pigments are rather scarce compared to inorganic white pigments, because (i) inorganic pigments such as CaCO_3_ or TiO_2_ are easy to synthesize industrially; also plants and animals can easily produce CaCO_3_ or other inorganic pigments; (ii) inorganic pigments generally have a much higher refractive index than organic pigments, leading to a better light scattering and to a better hiding power, *i.e.* they are of a more intense white. The hiding power depends on the difference of the refractive index between pigment and air, and on the particle size, which is optimal at around 0.5 µm. The wings of the *Pieris* butterflies contain small scales and empty spheres with a thickness of 0.7–2 µm surrounded by air. The beads have a very high refractive index, with values above 2 across the visible wavelength range, as determined by Jamin–Lebedeff interference microscopy combined with Kramers–Kronig theory and light-scattering modelling (Wilts *et al.*, 2017 ▸). However, it is not known why butterflies use leucopterin as a white pigment. Until today, the crystal structure and optical properties of leucopterin have been unknown.

**Figure 2:**
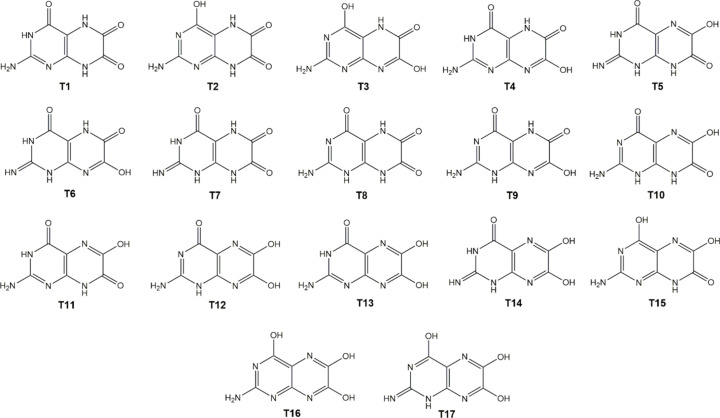
17 of the possible tautomers of leucopterin.

In principle, leucopterin can assume a large variety of tautomeric forms; some of them are shown in Fig. 2 ▸. The tautomeric state in solution was assessed by Pfleiderer and Rukwied in 1961. They observed that the UV spectrum of leucopterin in solution bears similarities with respect to the spectra of other substituted pteridines (Pfleiderer & Rukwied, 1961 ▸), and concluded that the solution should contain tau­to­mer T1.

The tautomeric form in the solid state can differ from that in solution, as it was shown in some cases, *e.g.* the one of the thermodynamically stable form of barbituric acid, which exhibits a keto form in solution, but the enol tautomer in the solid state (Schmidt *et al.*, 2011*a* ▸,*b* ▸; Marshall *et al.*, 2016 ▸). For leucopterin, 17 tautomers with a sensible chemical structure (without radicals or formal charges) could be drawn (see Fig. 2 ▸). The actual tautomeric form in the solid state was not known hitherto.

In this article, we report on the synthesis and crystal structure determination of leucopterin hemihydrate and its 0.2-hydrate using a combination of DTA-TG, tem­per­ature-dependent powder X-ray diffraction (PXRD), Fit with Deviating Lattice parameters (FIDEL) to the powder X-ray pattern, fit to the pair distribution function (PDF), single-crystal X-ray diffraction (SCXRD), Rietveld refinement, multinuclear solid-state NMR (SSNMR) and DFT-D optimization.

## 2. Experimental

### 2.1. Materials

2,4,5-Tri­amino-6-hy­droxy­py­rimi­dine sulfate was purchased from TCI. After more than 10 years of storage in the laboratory, the powder consisted of a mixture of the anhydrate (about 75%) and a monohydrate (about 25%), as revealed by powder X-ray diffraction (Tapmeyer & Prill, 2019 ▸).

Oxalic acid dihydrate was obtained from TCI.

### 2.2. Synthesis

2,4,5-Tri­amino-6-hy­droxy­py­rimi­dine sulfate (1.01 g, as a 3:1 mixture of anhydrate and monohydrate) was ground with oxalic acid dihydrate (2.65 g) and heated in a round-bottomed flask. At about 90 °C, a condensate began to appear. The pres­sure was reduced to about 100 mbar (1 bar = 10^5^ Pa) and the reaction mixture was heated to 140 °C until the reaction was complete. A yellow–brown product was obtained. For purification, this product was dissolved in a mixture of water (70 ml) and 2 *M* NaOH (2 ml), treated with activated carbon and filtered. The filtrate was poured into hot 2 *M* HCl (50 ml), which caused the leucopterin to pre­cipi­tate as a fine powder within several hours. The powder was isolated with a 2.7 µm filter, subsequently suspended in boiling water, filtered hot and dried on the filter under ambient conditions, yielding a pale-beige powder. In other syntheses, the final product was dried in a vacuum, or at ambient pressure and a tem­per­ature of 50 or 70 °C.

Elemental analysis after drying at 50 °C found (%, errors in brackets): C 35.18 (8), H 2.8 (3), N 35.8 (3); calculated for C_5_H_5_N_5_O_3_·0.5H_2_O: C 35.29, H 2.97, N 34.31, O 27.43.

Elemental analysis after heating to 250 °C in the differential thermal analysis (DTA) found (%, errors in brackets): C 34.8 (5), H 2.98 (5), N 35.5 (7); calculated for C_5_H_5_N_5_O_3_: C 36.92, H 2.59, N 35.89, O 24.60.

### 2.3. Differential thermal analysis and thermogravimetry (DTA-TG)

Differential thermal and thermogravimetric analysis (DTA-TG, Fig. S2 in the supporting information) were performed on a SETARAM (TGA 92) device. The samples were placed in Al_2_O_3_ crucibles and measured in a dynamic argon atmosphere with a heating rate of 10 °C min^−1^ and a constant flow rate of about 75 ml min^−1^.

### 2.4. Growth of single crystals of the hemihydrate

Single crystals could be obtained from a modified synthesis and purification procedure: 2,4,5-tri­amino-6-hy­droxy­py­rimi­dine sulfate (9.5 g, 0.05 mol) was ground with oxalic acid dihydrate (22 g, 0.2 mol) and heated in a round-bottomed flask to 140 °C. Over the course of 15 min, the tem­per­ature was increased to 170 °C and then within 15 min to 200 °C under a gentle vacuum. After maintaining the tem­per­ature at 200 °C for 30 min, the tem­per­ature was increased to 220 °C to remove excess oxalic acid. The residue was treated with 0.1 *M* NaOH (300 ml) and activated carbon at room tem­per­ature, and allowed to stand overnight. The carbon was removed by filtration, whereby the filtrate was directly poured into boiling 2 *M* HCl, which caused the product to pre­cipi­tate. The pre­cipi­tate, roughly dried by vacuum filtration, was again dissolved in 1 *M* NaOH (200 ml), filtered and pre­cipi­tated with 1.5 *M* HCl (200 ml), followed by drying at 70 °C. 109 mg of this product was added to a solution of 5.3 g Na_2_CO_3_ in 100 ml H_2_O, and again activated carbon (100 mg) was added. After briefly boiling, the mixture was filtered and the filtrate was poured immediately into boiling 1 *M* H_2_SO_4_ (100 ml), giving a clear yellow solution, in which tiny flat square-shaped crystals of leucopterin formed slowly. The crystals were washed with water and dried under ambient conditions.

### 2.5. Single-crystal structure analysis

A small thin square-shaped crystal with dimensions of about 20 × 15 × 2 µm was mounted on a glass pin (Fig. S3 in the supporting information) and measured on a Siemens/Bruker three-circle diffractometer equipped with an Incoatec microfocus source with mirror optics and an APEXII CCD detector. Cu *K*α radiation was used. Since the crystal was small and very thin, a prolonged counting time had to be employed, which led to an increased background. The data were scaled using *SADABS* (Sheldrick, 1996 ▸). The structure was solved by intrinsic phasing using *SHELXT* (Sheldrick, 2015*a* ▸) and refined by the full-matrix least-square method using *SHELXL* (Sheldrick, 2015*b* ▸). The data quality allowed only an iso­tropic refinement of all non-H atoms. Two restraints had to be applied to the *U*_iso_ values of the C atoms: the *U*_iso_ values of C01 and C08 were restrained to be equal, and the same was done for C09 with C10. H atoms were added after refinement, according to the tautomeric state determined by SSNMR and DFT-D.

The crystal was probably twinned. A twin refinement was tested but, due to the limited crystal quality, no conclusions could be drawn.

### 2.6. Powder X-ray diffraction

#### 2.6.1. Powder diffraction for phase analysis, structure solution and Rietveld refinement

Powder diffraction data were recorded at room tem­per­ature with Cu *K*α_1_ radiation in transmission mode on a Stoe Stadi-P diffractometer equipped with a primary Ge(111) monochromator and a linear position-sensitive detector (PSD).

For phase identification, the powders were prepared in glass capillaries of 0.7 mm diameter and a 2θ range of 2–60° was used.

For tem­per­ature-dependent PXRD, a sample which was dried under ambient conditions was prepared in an open 1.0 mm capillary, and measured in a flow of hot dry nitro­gen, using an Oxford Cryostream device in a 2θ range of 3–60° at tem­per­atures up to 226 °C.

For the Rietveld refinement of the 0.2-hydrate, leucopterin was heated to 250 °C in the DTA, cooled to room tem­per­ature and used to fill a 0.7 mm capillary. Data were collected with a PSD step size of 0.2° and a measurement time of 120 s per step. A 2θ range of 2.00–80.00° was collected with a resolution of 0.01°, resulting in 7800 data points.

For all PXRD measurements, the software *WinXPOW* (Stoe & Cie, 2005 ▸) was used for data collection and reduction.

#### 2.6.2. Synchrotron powder diffraction for PDF refinement

For the PDF refinements, different samples were used to fill polyimide capillaries of 1 mm diameter, which were measured with synchrotron radiation at the Diamond Light Source in Didcot, UK, at the beamline I15-1 using a Perkin­Elmer detector. The capillaries were rotated in a 10 Hz capillary spinner. A monochromatic incident X-ray beam was used with a standard size at the sample of 700 µm × 150 µm, conditioned using a bent Laue monochromator to provide an energy of 76 keV (λ = 0.1631 Å). The collected 2D synchrotron powder diffraction data were automatically converted to a 1D data set (Fig. S4 in the supporting information) using *DAWN* (Filik *et al.*, 2017 ▸).

#### 2.6.3. Obtaining the PDF

The pair distribution function G(*r*) (Fig. S5 in the supporting information) was obtained from the synchrotron powder data by background correction, normalization and Fourier transformed using the program *PDFgetX3* (Juhás *et al.*, 2013 ▸). The data were truncated at a finite maximum value of the momentum transfer *Q*_max_ = 20.3 Å^−1^.

### 2.7. FIDEL fits for structure determination from powder data (SDPD) of the 0.2-hydrate

FIDEL provides a method to fit a molecular crystal structure to an experimental powder pattern, even if the lattice parameters do not match. FIDEL uses a cross-correlation function to compare simulated and experimental powder patterns (Habermehl *et al.*, 2014 ▸, 2022 ▸).

Here, FIDEL was employed for two tasks: (i) the refinement of the structure of the 0.2-hydrate starting from the structure of the hemihydrate in *P*2/*c*; (ii) a ‘regional fit’, *i.e.* a global optimization with FIDEL-GO starting from a large number of random crystal structures in *P*2_1_/*c* within a given range of lattice parameters, but with random position and orientation of the molecules.

#### 2.7.1. FIDEL fit in *P*2/*c*

The fit started from the single-crystal data of the hemihydrate, including H atoms. The water molecule was removed. For the FIDEL fit, a 2θ range of 4–40° was used. In FIDEL, the default settings of all parameters were used.

#### 2.7.2. Regional FIDEL fit in *P*2/*c*

The calculations started from a large set of random structures in *P*2_1_/*c*, *Z* = 4, with a restricted range for the lattice parameters. Random starting values were used for the molecular positions and orientations. After removing the structures with overlapping molecules, about 2 million structures remained. These structures were fitted to the experimental powder data using the cross-correlation function in an elaborated automated multi-step process, which includes ranking according to the similarity to the powder pattern, clustering and removal of duplicates (Habermehl *et al.*, 2022 ▸). The best structures had a similarity index much worse than the structure in *P*2/*c*.

### 2.8. Rietveld refinement of the 0.2-hydrate

The structure from the FIDEL fit in *P*2/*c* was subjected to an automated Rietveld refinement with FIDEL calling *TOPAS*, as described by Habermehl *et al.* (2014 ▸). Subsequently, a manually controlled Rietveld refinement was performed with *TOPAS Academic 4.2* (Coelho, 2018 ▸). A 2θ range of 4–70° was used. The background was modelled using a Chebychev polynomial with 20 parameters. Further refined parameters included the peak profile, zero-point shift, lattice parameters, all atomic coordinates, including H-atom positions, and one overall iso­tropic displacement parameter. The aniso­tropic peak broadening was modelled by spherical harmonics of the fourth order. Restraints were applied for all bond lengths, bond angles and for the planarity of the entire molecule. Preferred orientation was neither observed nor refined.

### 2.9. Structure solution attempts by global fit to the PDF

Attempts for determining the structure by a fit to the pair distribution function (PDF) from scratch without prior indexing were performed as described by Schlesinger *et al.* (2021 ▸). Calculations were run in various space groups, each with *Z*′ = 1. Simulated and experimental PDFs were compared in the range 1–30 Å. The molecules were treated as rigid bodies, only refining their positions and orientations. Additionally, the zero point, a global scale factor, lattice parameters, two *B*_iso_ values and a dampening factor were refined.

### 2.10. Lattice-energy minimizations by DFT-D

Geometry optimizations of 17 of the possible tautomers of leucopterin were performed in the gas phase with *GAUSSIAN09* (Frisch *et al.*, 2009 ▸) with the classical B3LYP functional coupled with the optimized def2-SVP basis set and the D3 version of Grimme’s dispersion method, including the Becke–Johnson damping scheme.

The X-ray structures of the hemihydrate and the anhydrate (starting from the X-ray structure of the hemihydrate, but omitting the water molecules) were optimized employing the *Quantum ESPRESSO* suite (Version 6.4.1; Giannozzi *et al.*, 2009 ▸), using the projector augmented wave (PAW) approach, the non-local vdW-DF2 method (Lee *et al.*, 2010 ▸) and the B86r functional of Hamada (2014 ▸), with the efficient set of standard solid-state pseudopotentials (Prandini *et al.*, 2018 ▸; Lejaeghere *et al.*, 2016 ▸). An energy cut-off of 60 Ry was used. For each model, two calculations were run in two subsequent steps: in the first step, the lattice parameters were taken from the Rietveld refinement and kept fixed, optimizing only the atomic positions. The resulting structure was further optimized including also the lattice parameters. Periodic plane wave calculations were repeated for each of the 17 tautomers. Initial structures were obtained from the experimental hemihydrate, by correctly positioning the H atoms according to tautomer formulae (Fig. 2 ▸). A second non-local vdW-DF2 method adopting the optB88 gradient correction to exchange (which uses the Becke88, B88, exchange) (Becke, 1988 ▸; Klimeš *et al.*, 2011 ▸) was also performed.

### 2.11. Calculation of SSNMR spectra

On the basis of the structures optimized by *Quantum ESPRESSO*, the SSNMR calculation was performed using the Gauge Including Projected Augmented Wave (GIPAW) (Pickard & Mauri, 2001 ▸) and the PBE pseudopotentials from PS Library 1.0.0 (Corso, 2014 ▸) with an energy cut-off of 80 Ry, which is a reasonable compromise between accuracy and efficiency (Wolczyk *et al.*, 2017 ▸). The obtained absolute iso­tropic magnetic shielding (σ_iso_) values were converted into iso­tropic chemical shifts (δ_iso_) relative to the absolute mag­netic shielding of 1,4-di­aza­bi­cyclo[2.2.2]oc­tane (DABCO), used as a reference substance, applying Equation 1.

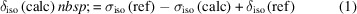

The δ_iso_(ref) values of DABCO are 0.00, 47.74 and 10.97 ppm for ^1^H, ^13^C and ^15^N, respectively, while the absolute iso­tropic constant shieldings σ_iso_(ref) are 27.03, 120.59 and 212.22 ppm for ^1^H, ^13^C and ^15^N, respectively, calculated from the reported neutron diffraction crystal structure (Nimmo & Lucas, 1976 ▸).

### 2.12. SSNMR measurements

The solid-state ^13^C and ^15^N CPMAS spectra were acquired with a Jeol ECZR 600 instrument (14.1 T), operating at 600.17, 150.91 and 60.83 MHz, respectively, for ^1^H, ^13^C and ^15^N nuclei.

The powder samples were packed into a cylindrical zirconia rotor with a 3.2 mm outer diameter (60 µl volume) and spun at 20 (^1^H and ^13^C) and 12 kHz (^15^N). A certain amount of sample was collected from each batch and used without further preparations to fill the rotor. The ^13^C and ^15^N CPMAS spectra were acquired at room tem­per­ature using a ramp cross-polarization pulse sequence with a 90° ^1^H pulse of 2.1 µs and contact times of 3.5 (^13^C) and 0.1–4 ms (^15^N). Optimized recycle delays of 6 and 12 s were used for numbers of scans of 1100 (^13^C) and 3000–10000 (^15^N). For every spectrum, a two-pulse phase modulation (TPPM) decoupling scheme was used, with a radiofrequency field of 108.5 kHz.

The solid-state 1D ^1^H and 2D ^1^H DQ MAS (double-quantum magic angle spinning) spectra were acquired with a Bruker Ascend instrument (23.2 T), operating at 1000.40 MHz for ^1^H nucleus and equipped with an Avance NEO console.

The powder samples of the hemihydrate and ‘anhydrate’ (which turned out to contain traces of water) were packed into a cylindrical zirconia rotor with a 0.7 mm outer diameter and spun at 100 kHz. The ‘anhydrate’ sample was packed in a glove-box. 1D ^1^H spectra were acquired with a 90° ^1^H pulse of 2.75 µs and optimized recycle delays of 16 (T_1_ of 12 s for the hemihydrate) and 40 s (T_1_ of 32 s for the ‘anhydrate’) were used for 16 transients.

For the ^1^H DQ MAS, one rotor period of the back-to-back (BaBa) (Bradley *et al.*, 2009 ▸; Brown *et al.*, 2004 ▸; Brown, 2012 ▸) recoupling sequence was used for the excitation and reconversion of DQ coherences. A 16-step phase cycle was used in order to select Δ*p* = ±2 on the DQ excitation pulses (4 steps) and Δ*p* = −1 (4 steps) on the z-filter 90° pulse, where *p* is the coherence order. These experiments were acquired with a 90° ^1^H pulse of 2.75 µs and 16 transients were co-added for each of 128 t1 FIDs, using the States-TPPI method to achieve sign discrimination in F1 with a t1 increment of 25 µs.

The ^13^C chemical shift scale was calibrated through the methyl­enic signal of external standard α-glycine (at 43.7 ppm); the ^15^N chemical shift scale was calibrated through the signal of external standard α-glycine (at 33.4 ppm with reference to liquid NH_3_). The ^1^H chemical shifts obtained at the 1 GHz instrument at the High Field National facility were calibrated using as external reference DSS [3-(tri­methyl­silyl)-1-pro­pane­sulfonic acid-*d*_6_ sodium salt] at 0 ppm.

## 3. Results and discussion

### 3.1. Leucopterin: a variable hydrate

Leucopterin was synthesized according to Scheme 2, reproducing the synthesis of Purrmann (1940 ▸). Chemical analysis, as well as Debye–Scherrer diagrams, confirmed that the synthesized product corresponds to leucopterin isolated from butterfly wings in 1933. Also, the crystal phase is the same (see Fig. S1 in the supporting information).

**Figure 3:**
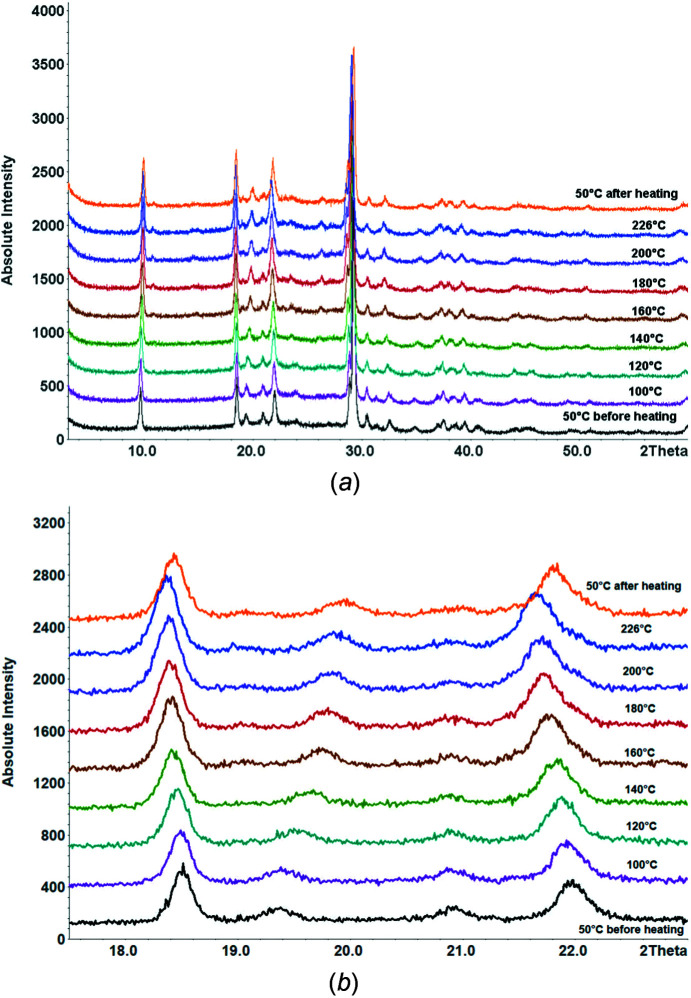
(*a*) Temperature-dependent powder patterns of leucopterin; (*b*) magnification of the tem­per­ature-dependent powder patterns of leucopterin in the region 17.5–23.0° (2θ).

**Figure 4:**
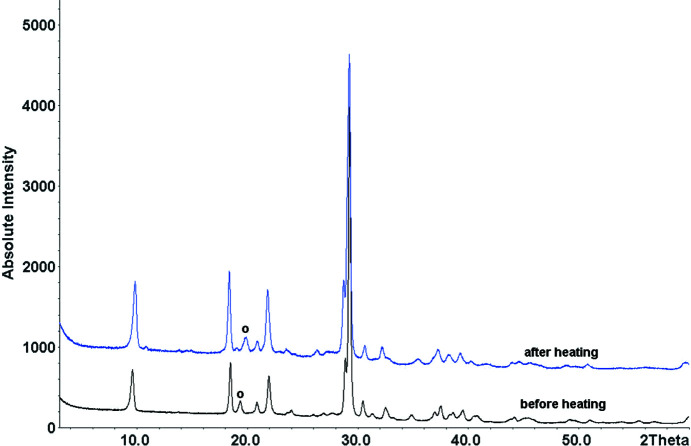
Powder patterns of leucopterin before (black) and after heating (blue) to 250 °C. Both patterns were measured at room tem­per­ature. The circle denotes one of the weak reflections which shifted upon dehydration.

The PXRD patterns of different synthetic samples looked visually quite similar, but some peaks had varying peak positions and intensities. Temperature-dependent PXRD showed a continuous peak shift from 50 to 226 °C. After cooling to room tem­per­ature, the peaks remained shifted (Fig. 3 ▸), which shows that the peak shift upon heating was not only a tem­per­ature effect, but corresponded to a change of the sample itself. Fig. 4 ▸ shows the powder pattern before and after heating to 250 °C.

The DTA-TG of leucopterin hemihydrate (Fig. S2 in the supporting information) showed a slow mass loss of 3.5% between 130 and 250 °C, corresponding to the release of 0.4 water molecules per leucopterin. Upon further heating, no additional weight loss was observed up to decomposition, which occurred at about 410 °C.

Consequently, leucopterin is a variable hydrate with a water content between 0.5 and about 0.1 molecules.

It remains uncertain if it is possible to remove all water from this phase, *i.e.* if the phase width of this variable hydrate includes the anhydrate, too. We never obtained a sample which was definitively an anhydrate, *i.e.* completely free of water molecules. The TG of the hemihydrate showed a release of 0.4 instead of 0.5 molecules of water, but this difference is within experimental uncertainty. The SSNMR samples of the ‘anhydrate’ always contained traces of water (but this might be a result of the sample preparation, since the hemihydrate is more stable). The Rietveld refinement of a sample heated to 250 °C in the DTA and cooled to room tem­per­ature led to a value of 0.2 molecules of water, but this value, determined only from the occupancy of the water O atom in the Rietveld refinement, is uncertain, too. A virtually identical PXRD pattern was observed in a slow measurement at 220 °C of a sample in an open capillary in a flow of hot dry nitro­gen. Under these conditions, one should expect the formation of an anhydrous sample, but a final proof of an anhydrate is still missing.

### 3.2. Attempts for crystal growth

The solubility of leucopterin in water and other organic solvents (methanol, tetrahydrofuran, dioxane, pyridine, *N*-methyl­pyrrolidone, nitro­benzene, chloro­benzene, di­chloro­benzene, 1,2,4-tri­chloro­benzene, toluene and benzene) is lower than 0.5 g l^−1^. Crystal growth from the melt is not possible because leucopterin does not melt below 410 °C, at which tem­per­ature it decomposes. Attempts at sublimation failed as well.

### 3.3. Attempts to solve the crystal structure by PXRD

Attempts at indexing the PXRD patterns of different samples led to different orthorhombic or monoclinic unit cells with volumes of about 710 Å^3^ (or multiples of it) and monoclinic angles close to 90°. The systematic extinctions (as far as visible from the powder data) pointed to the space groups *P*2/*c*, *Pc*, *P*2_1_/*m*, *P*2_1_2_1_2_1_, *Pcca*, *Pbam* or *Pbca*. Attempts to solve the crystal structure by the direct-space method using *DASH* (David *et al.*, 2006 ▸) resulted in various solutions. However, the subsequent Rietveld refinement failed: the fits were poor and most of the structures were crystallochemically senseless. After we had solved the structures by other methods (see below), we recognized that our attempts actually contained trials with the correct lattice parameters (*a* ∼ 8.1 Å, *b* ∼ 4.8 Å, *c* ∼ 18.3 Å and β ∼ 90.2°) and with the correct space group (*P*2/*c*), but with the wrong space group setting (*P*2/*a* instead of *P*2/*c*), which was the reason why the structure determination from powder data failed.

### 3.4. Attempts to solve the crystal structures by global fits to the pair distribution function (PDF)

The PDF represents the probability of finding pairs of atoms with an interatomic distance *r*, and it is weighted by the scattering power of the corresponding atoms and normalized to a homogeneous atom density. Experimentally, the PDF is the Fourier transform of the intensity profile of a powder pattern. Correspondingly, it contains, in principle, the same information as the powder pattern itself, but the information is provided in the direct space instead of the reciprocal one. The PDF describes the actual local structure (similar to SSNMR), whereas the Bragg reflections reflect the long-range order.

In recent years, we developed a method to solve and refine organic crystal structures by a global fit to the PDF using the program FIDEL. The method also works if the lattice parameters are not known. In this case, the PDF fits start from a huge number of random crystal structures with random lattice parameters in various space groups (PDF-Global-Fit; Prill *et al.*, 2016 ▸; Habermehl *et al.*, 2021*a* ▸,*b* ▸; Schlesinger *et al.*, 2021 ▸).

This method was also applied to leucopterin. Samples with different water contents were measured with short-wavelength synchrotron radiation (λ = 0.1631 Å), so that PDFs with a good resolution were obtained. Structure solution attempts were carried out in various space groups, each with *Z*′ = 1 for leucopterin and without water. In some of the PDF global fit series, the starting values for the lattice parameters were fully random; in other trials, the starting values were set to *a* ∼ 18.3 Å, *b* ∼ 4.8 or 9.6 Å, *c* ∼ 8.1 Å, α = γ = 90° and β ∼ 90.3 (for monoclinic space groups) or β = 90° (for orthorhombic space groups). The PDF global fits worked well and yielded several different crystal structures with quite a good fit to the experimental PDF [the best fit is shown in Fig. S6(*a*) in the supporting information]. However, their simulated powder patterns did not match the experimental ones. Hence, these structures represent fairly good models for the local structure, but not for the long-range ordering.

**Figure 5:**
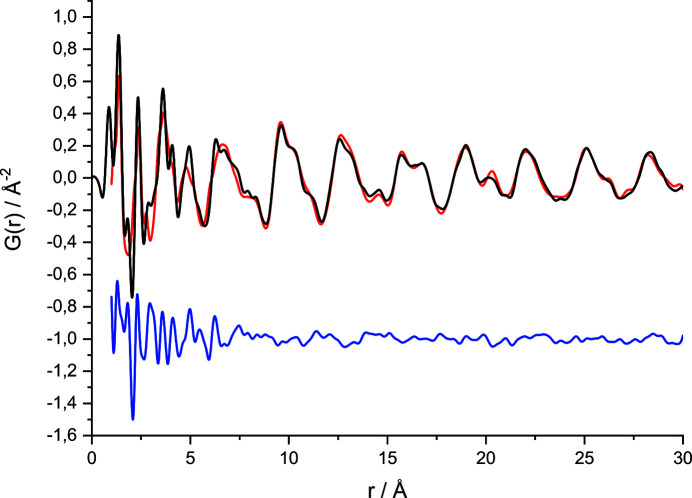
Successful crystal structure determination of leucopterin hemihydrate by a global fit to the PDF, performed after the correct space group (*P*2/*c*) was known. The PDF global fit was done without the water molecule. The experimental PDF is shown in black, the calculated PDF in red and the difference curve below in blue.

After the structures of the hemihydrate and the 0.2-hydrate were solved by other methods, it turned out that the assumed lattice parameters were actually correct, but the PDF fits were not performed in the correct space group *P*2/*c*; hence, they were not able to yield the correct long-range order. A later PDF global fit in *P*2/*c*, *Z* = 4, resulted in the correct structure. The agreement between simulated and experimental PDF (Fig. 5 ▸) was much better than in all other trials. The *R* value was much lower and the resulting crystal structure was virtually identical to the single-crystal structure (see Fig. S6 in the supporting information). Hence, the crystal structure of leucopterin hemihydrate could have been successfully solved by a PDF fit without any problems, if only we had included the correct space group.

### 3.5. Single-crystal X-ray diffraction of the hemihydrate

Finally, we obtained tiny crystals. The crystals were very thin small platelets with a size of about 30 µm and a thickness of only about 2 µm (see Fig. S3 in the supporting information). Correspondingly, the reflection intensities were weak and the quality of the data set was poor. The indexing led to a monoclinic unit cell with *a* ∼ 8.1 Å, *b* ∼ 4.8 or 9.6 Å, *c* ∼ 18.3 Å and β ∼ 90.2°. Also, orthorhombic metrics could not be ruled out. The unit-cell volumes of about 710 or 1420 Å^3^ pointed to *Z* = 2.7 or 5.4, according to the ‘18 Å rule’ (Kempster & Lipson, 1972 ▸), and to *Z* = 3.3 or 6.6 using the volume increments of Hofmann (2002 ▸). All these values are strange for monoclinic and orthorhombic structures. The extinction rules were difficult to assess. The extinction symbol seemed to be *P*–*c*–, but the space group remained ambiguous. Structure solution and refinement were performed in various space groups, including *P*2_1_ (*Z* = 8), *Pc* (*Z* = 4 or 8), *P*2/*c* (*Z* = 4 or 8), *P*2_1_/*c* (*Z* = 8), *P*2/*m* (*Z* = 4), *Pmc*2_1_ (*Z* = 4) and *Pcc*2 (*Z* = 4). In all structural models, half a water molecule per leucopterin molecule was found, *i.e.* all structures were hemihydrates. The best results were obtained in *P*2/*c*, *Z* = 4, with *wR*2 = 14.5%, whereas all other refinements lead to *wR*2 values of more than 32.0%.

Due to the poor data quality, the H atoms could not be located. The accuracy of the C—C, C—N and C—O bond lengths was too low to distinguish between single and double bonds. Correspondingly, the tautomeric state remained unclear. Therefore, this was determined by SSNMR and DFT-D (see below), and the H atoms were finally added in calculated positions. The final crystallographic data are given in Table 1 ▸, while a comparison between the unit-cell parameters of the optimized and experimental crystal structures is reported in Table 2 ▸.

The powder pattern simulated from the single-crystal data agreed very well with the experimental powder patterns of other samples, which were also obtained from water and dried under ambient conditions, proving that leucopterin exists as a hemihydrate under ambient conditions.

### 3.6. Structure determination of leucopterin 0.2-hydrate from powder data

#### 3.6.1. Structure solution by Fit with Deviating Lattice parameters (FIDEL)

The powder patterns of the variable hydrate are similar within the entire range, which indicates that the water content has only a minor effect on the overall crystal structure.

Thus, we started from the structure of the hemihydrate and removed the water. As seen from Fig. 3 ▸, some peaks shifted considerably upon the dehydration of the hydrate. In a Rietveld refinement, the experimental and simulated powder patterns are compared point-by-point for each 2θ value. Rietveld refinements generally do not work when the lattice parameters deviate significantly. For this task, we developed FIDEL fit, which uses a cross-correlation function for comparing experimental and simulated powder patterns, and has a much longer convergency range.

We used the powder X-ray pattern of a sample which was previously dehydrated at 250 °C and subsequently measured at room tem­per­ature. The FIDEL fit in *P*2/*c* was very convincing and resulted in a good fit of all peaks, and good similarity values between simulated and experimental powder patterns.

#### 3.6.2. Confirmation of the space group by a global FIDEL fit

In *P*2/*c*, the missing water molecules leave a void. Furthermore, *P*2/*c* is a very rare space group for homomolecular organic compounds (*i.e.* crystals of pure compounds not containing ions, solvents or water molecules): only 0.05% of structures crystallize in *P*2/*c*, *Z* = 4, with molecules on general positions (Belsky *et al.*, 1995 ▸). The *P*2_1_/*c* symmetry with *Z* = 4 is 500 times more frequent. In the structure of leucopterin, a transition from *P*2/*c* to *P*2_1_/*c* would require only a molecular shift by 1.2 Å in the *b* direction, which would easily be possible during dehydration. Therefore, we additionally searched for a structure solution in *P*2_1_/*c*, *Z* = 4. Also for this task, the FIDEL program was used. The fit started from 2 million random crystal structures with random lattice parameters. These structures were fitted to the powder pattern of the dehydrated sample resulting in 78 promising structures. However, all these structures gave a worse fit than that in *P*2/*c*, thereby ruling out the space group *P*2_1_/*c*.

#### 3.6.3. Rietveld refinement

**Figure 6:**
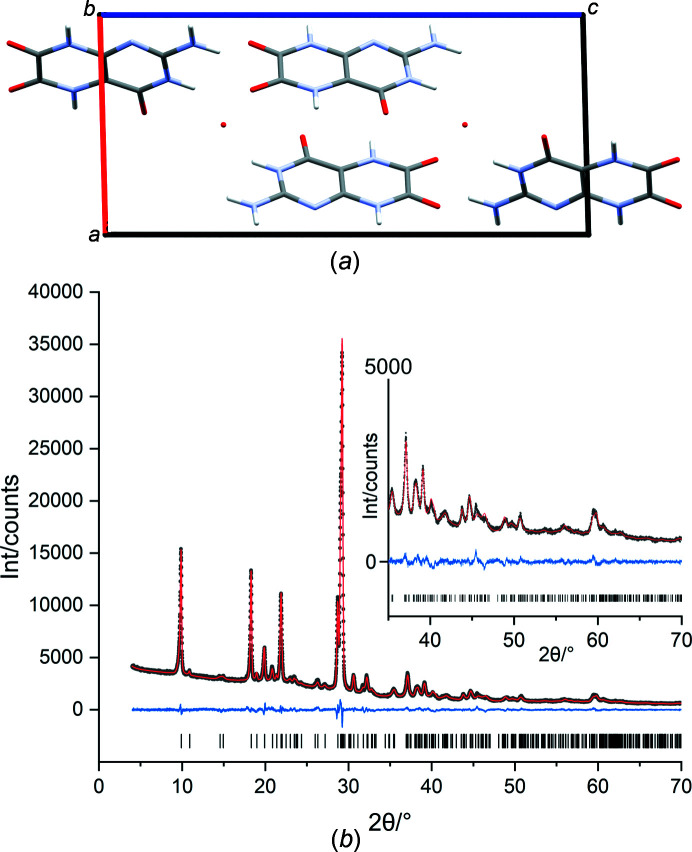
(*a*) Crystal structure of leucopterin 0.2-hydrate from Rietveld refinement; the view direction is [0 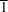 0]. The red points indicate the position of the water molecules refined as an O atom with an occupancy of 0.42. (*b*) Rietveld plot of leucopterin 0.2-hydrate. The experimental pattern is drawn with black points, the calculated pattern as a red line and the difference curve in blue. The vertical tick marks denote the reflection positions.

The *P*2/*c* structure of the anhydrate from the initial FIDEL fit was subjected to an automatic Rietveld refinement performed by FIDEL-called *TOPAS*, followed by a manually controlled Rietveld refinement. The refinement converged to quite a good fit and con­fidence values of *R*_p_ = 2.88, *R*_wp_ = 3.97 and GOF = 1.83. Finally, we tested if the investigated powder still contained some water molecules in the lattice. For this task, an O atom was placed in the former water position and the structure was refined once more. The *R*_wp_ value dropped from 4.0 to 3.5 and the GOF from 1.8 to 1.6. The crystal structure and the Rietveld plot are reported in Figs. 6 ▸(*a*) and 6 ▸(*b*). The occupancy of the O atom was refined to 0.421 (8), which corresponds to about 0.17 water molecules per leucopterin. Hence, according to the Rietveld refinement, this structure should be regarded as a hydrate with 0.1 to 0.2 molecules of water per leucopterin molecule. However, the refinement of the occupancy of a single O atom is not reliable for powder data of this quality.

To check whether the structure was correct, we performed a refinement without restraints, using fixed positions for the H atoms. All *R* values got better, but the molecule became strongly distorted, as can be seen in Fig. S7 in the supporting information. The maximum shift of the C atoms was 0.7 Å (for C2).

The occupancy of the water O atom dropped from 0.42 to 0.213 (11), corresponding to 0.085 (4) water molecules per leucopterin. Hence, the structure is in principle correct, but the low quality of the powder data did not allow for a precise determination of the atomic coordinates. It is highly uncertain if the structure indeed contains water molecules in the lattice.

### 3.7. SSNMR analysis: determination of the tautomeric state

To assess the tautomeric character of leucopterin hemihydrate and anhydrate, several multinuclear 1D SSNMR experiments (^1^H MAS, ^13^C and ^15^N CPMAS) and 2D ^1^H DQ MAS SSNMR experiments were performed. Table 3 ▸ contains the experimental ^1^H, ^13^C and ^15^N chemical shifts with assignments, as well as the shifts computed with the optB88 method. ^1^H, ^13^C and ^15^N chemical shifts computed with the B86r method are reported in Tables S1 and S2 in the supporting information.

The experiments revealed that the investigated ‘anhydrate’ sample actually contained traces of water. Together with the PXRD data, this confirms that the ‘anhydrate’ is actually a variable hydrate. However, PXRD provides only an averaged global description of the structure, whereas SSNMR is a local structural method. This allows for observing how, in the variable hydrate, leucopterin molecules attached to a water molecule give signals typical of the hemihydrate, whereas leucopterin molecules without a water molecule in their vicinity give signals potentially coincident with those of the real anhydrate. Hence, SSNMR is able to provide information on the tautomerism of leucopterin in its anhydrate state, although the investigated sample was not completely water-free. In the following description, we use the description ‘anhydrate’ for the dehydrated sample still containing traces of water.

**Figure 7:**
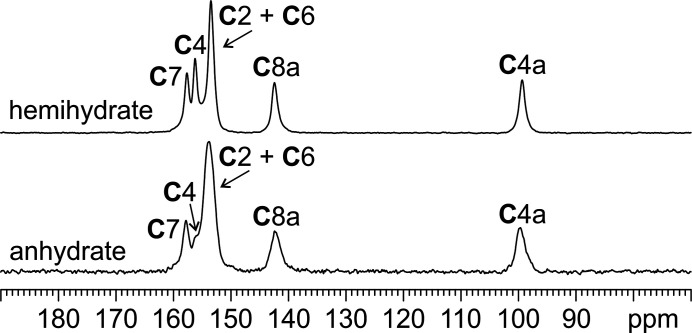
^13^C (150.9 MHz) CPMAS spectra of leucopterin hemihydrate and anhydrate, acquired at room tem­per­ature at a spinning speed of 20 kHz.

Fig. 7 ▸ displays the ^13^C CPMAS SSNMR spectra of leucopterin hemihydrate and anhydrate, referred to in the figures just as hemihydrate and anhydrate, respectively.

The ^13^C spectrum of the hemihydrate displays five sharp resonances [average full width at half maximum (FWHM) = 175 Hz], of which one, at 153.4 ppm, accounts for two nuclei.

The ^13^C signals of the anhydrate occur at very similar chemical shifts with respect to the hemihydrate, but they appear slightly broader (average FWHM = 243 Hz), especially that at 157.8 ppm. Nonetheless, five characteristic resonances can be distinguished for the anhydrate too, with that at 154.1 ppm accounting for two nuclei.

The ^15^N CPMAS spectra of the hemihydrate and anhydrate are also very similar (Fig. S8 in the supporting information). Five distinct resonances can be observed, each referred to the different five nitro­gen nuclei in leucopterin.

The high similarity between the SSNMR spectra of leucopterin hemihydrate and anhydrate strongly suggests that they possess the same chemical environment in terms of tautomeric character and local molecular arrangement and, thus, that the crystal structure does not significantly change upon dehydration.

**Figure 8:**
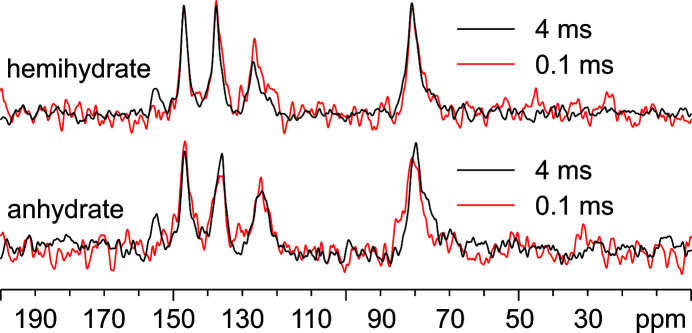
Overlays of the ^15^N (60.8 MHz) CPMAS (12 kHz) spectra at room tem­per­ature of leucopterin hemihydrate and anhydrate acquired with different contact times to highlight signals due to protonated and non-protonated N-atom sites. Top: hemihydrate, acquired with contact times of 0.1 (red) or 4 ms (black); bottom: anhydrate, acquired with contact times of 0.1 (red) or 4 ms (black).

To gain a qualitative insight into the number of hydrogenated N atoms in the two crystal forms, the ^15^N CPMAS spectra were acquired twice, *i.e.* with a very short (0.1 ms) and with a long (4 ms) contact time (Fig. 8 ▸). The former spectra (red in Fig. 8 ▸) highlight only the signals due to protonated signals since the proton magnetization is transferred only to very close ^15^N nuclei (*i.e.* covalently bonded to hydrogen) *via* heteronuclear dipolar interaction. The latter (black in Fig. 8 ▸) are characterized by resonances due to both protonated and non-protonated ^15^N nuclei, since the magnetization is transferred through spin diffusion during the long contact time.

Interestingly, for both hemihydrate and anhydrate, the signals at higher frequencies (154.9 ppm) vanish if a very short contact time (0.1 ms) is employed, indicating that the corresponding N atom does not carry H atoms. Furthermore, the signals at 79.9 ppm are consistent with the presence of amino (–NH_2_) groups rather than imino (=NH) groups, whose chemical shifts are higher, ranging from 170 to 300 ppm (Marek *et al.*, 2007 ▸; Derenne *et al.*, 2012 ▸).

**Figure 9:**
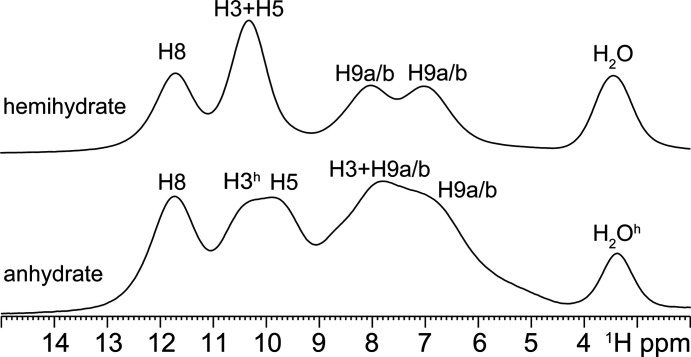
^1^H (1000.4 MHz) MAS spectra of leucopterin hemihydrate (above) and anhydrate (below), acquired at room tem­per­ature at a spinning speed of 100 kHz. H3^h^ and H_2_O^h^, in the anhydrate spectrum, refer to H3 and H_2_O signals originating from the amount of partially hydrated leucopterin.

**Figure 10:**
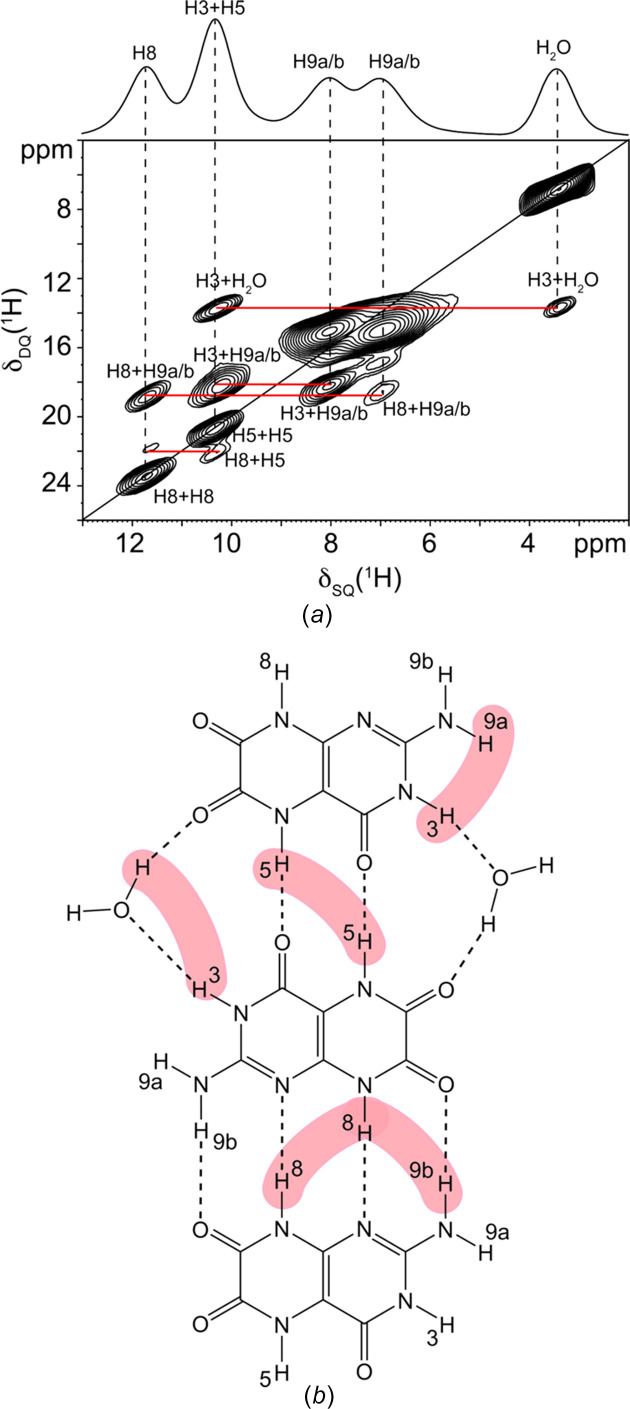
(*a*) ^1^H (1000.4 MHz) DQ MAS spectrum of leucopterin hemihydrate acquired at room tem­per­ature at a spinning speed of 100 kHz. Only the most relevant correlations are highlighted. (*b*) Scheme of the main H–H proximities observed in the ^1^H DQ MAS spectrum. The H3–H3 correlation is not shown since it is an interlayer proximity.

**Figure 11:**
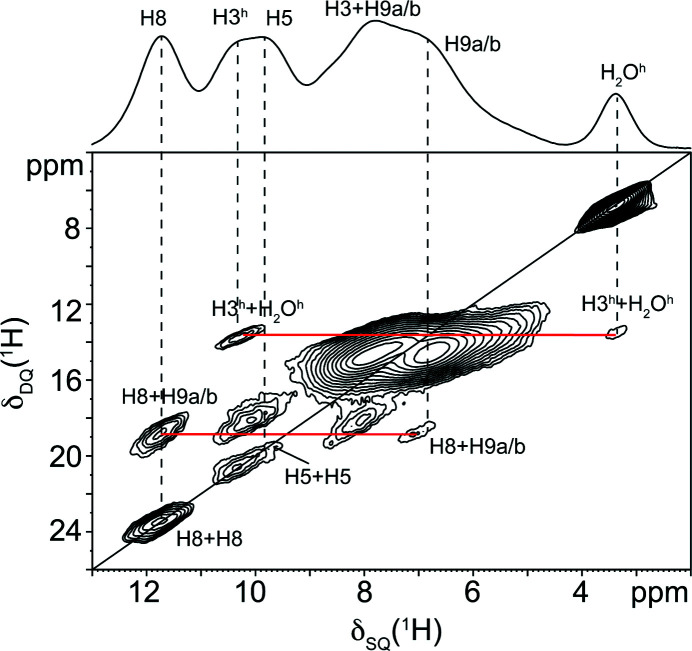
^1^H (1000.4 MHz) DQ MAS spectrum of leucopterin anhydrate acquired at room tem­per­ature at a spinning speed of 100 kHz. Only the most relevant correlations are highlighted. H3^h^ and H_2_O^h^, in the anhydrate spectrum, refer to H3 and H_2_O signals originating from the amount of partially hydrated leucopterin.

By combining this information, the SSNMR data points towards the presence of tautomer T1 or T8 for both leucopterin hemihydrate and anhydrate. Indeed, the T1 and T8 tautomers are the only two characterized by three NH sites, one unprotonated N and one NH_2_ group. To unambiguously assign the correct tautomer, ^1^H MAS and ^1^H DQ MAS experiments were performed. The ^1^H MAS spectra of the hemihydrate and anhydrate greatly benefited from the ultrafast spinning speed and high magnetic field since they display five relatively narrow resonances, as shown in Fig. 9 ▸. In the ^1^H DQ MAS of the hemihydrate [Fig. 10 ▸(*a*)], the chemical shifts that allowed the assignment of the distinct resonances had the typical values of amides (–NH) and amines (–NH_2_), and the specific H8–H8, H5–H5, H3–H3, H3–H_2_O, H3–H9a/b and H8–H9a/b correlations were detected. In the anhydrate sample, we were able to detect the incomplete dehydration of the sample, as shown by the presence of the residual H_2_O peak (3.4 ppm, marked as H_2_O^h^ in Figs. 9 ▸ and 11 ▸) in the ^1^H MAS spectrum and by the H3–H_2_O protons correlation (Fig. 11 ▸) typical of the hemihydrate form, suggesting the presence of a partially hydrated sample.

Although the ^1^H MAS spectra were not acquired under quantitative conditions (*i.e.* employing a relaxation delay ≥ 7T_1_), a rough estimation of the amount of water molecules per leucopterin molecule was performed, by integrating the signals in the spectra (see Fig. 9 ▸). This led to a value of 0.5 water molecules per leucopterin molecules in the hemihydrate, and 0.33 for the ‘anhydrate’.

The difference between the T1 and T8 tautomers is only in the H3/H1 position (peak at 10.4 ppm): in the case of the T8 tautomer, if the peak at 10.4 ppm was assigned to H1, we would detect the H1–H_2_O and H8–H1 correlations, which should be very intense because of the short distances between them. Instead, the visible weak correlation between H8 and the proton at 10.4 ppm is compatible with an H8–H5 interlayer correlation, given the long distance between them; at the same time, the intense cross-peaks at 10.4 and 3.4 ppm agree very well with the H3–H_2_O proximity. Considering all the NMR measurements and for all of the abovementioned reasons, tautomer T1 was assessed to be the one present in our samples. The other detectable correlations fit perfectly with the molecules alternatingly connected by two and four hydrogen bonds, visible also in the leucopterin structure. By analogy, the ^1^H assignments and correlations in the ^1^H DQ MAS of the anhydrate form (Fig. 11 ▸) indicate that it is characterized by tautomer T1.

### 3.8. DFT-D: determination of the tautomeric state and confirmation of the crystal structure

The tautomeric form of leucopterin in the crystal structure of leucopterin hemihydrate could not be assessed by SCXRD. Hence, it remained unclear which of the 17 tautomeric forms with reasonable chemical formulae (Fig. 2 ▸) exists in the solid state. The molecular stability of the tautomers was firstly investigated in the gas phase by *GAUSSIAN09* calculations (Frisch *et al.*, 2009 ▸). Out of the 17 tautormers, T1 and T2 displayed the lowest energy, while the others showed a higher relative energy, equal to or above 9 kJ mol^−1^ (Table S3 in the supporting information).

The relative energies of the tautomers in the solid state follow similar trends. Using *Quantum ESPRESSO*, we per­formed the geometry optimizations of the 17 crystal structure models, each containing a different tautomeric form. These models were built starting from the SCXRD data of leucopterin hemihydrate and localizing the H atoms according to formulae 1–17 sketched in Fig. 2 ▸. The main aim was not to assess the most stable solid-state structure of each tautomer, but which tautomer is thermodynamically more stable in the symmetry framework of the found experimental structure. Full optimizations were performed by two non-local vdW-DF2 DFT-D methods, the first adopting the B86r approach and the second adopting the optB88 method (see *Experimental*). All the optimized structures retained the space group *P*2/*c*. Relative energies, unit-cell parameters and ^1^H, ^13^C and ^15^N RMSEs (root mean squared errors) between the experimental and computed chemical shifts for T1 and T8 are reported in Table 4 ▸. The complete results are reported in Table S3 in the supporting information.

Leucopterin T1, *i.e.* the structural model containing tautomer T1, is by far the most thermodynamically stable structure, with the second best, *i.e.* T2, 62.29 kJ mol^−1^ (optB88) less stable. T1 also shows the smallest ^1^H, ^13^C and ^15^N RMSE values. Other tautomers should be excluded, not only because they display much higher energies, but also because their respective structural models exhibit substantially larger ^1^H, ^13^C and ^15^N RMSE values. In conclusion, the lowest energy, the very good agreement between the calculated and experimental solid-state structures, and the chemical shift calculations strongly point toward T1 as the tautomer present in the crystal structure of leucopterin hemihydrate.

### 3.9. Crystal structure of leucopterin hemihydrate

**Figure 12:**
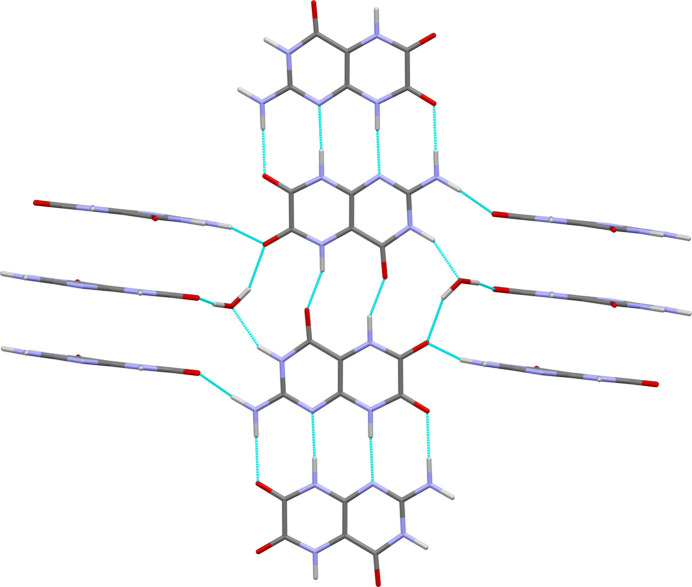
Molecular chains in the crystal structure of leucopterin hemihydrate, after DFT-D optimization with fixed lattice parameters. Colour code in all drawings: C = grey, O = red, N = blue, H = white and hydrogen bonds = turquoise. The view direction is [120].

In the crystal structure of leucopterin hemihydrate, the molecules show a planar conformation. They are on general positions, while the water molecules are located on twofold axes. The crystal is built from symmetrically equivalent planar leucopterin chains. Within the chains, the molecules are alternatingly connected by two and four hydrogen bonds (see Fig. 12 ▸).

**Figure 13:**
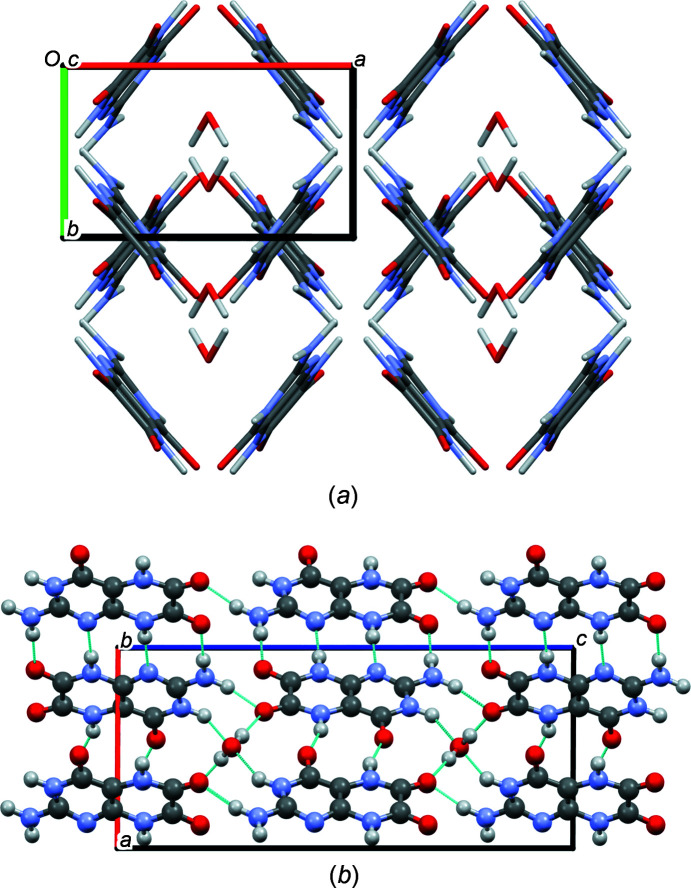
(*a*) Crystal packing (view direction [00 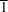 ]) and (*b*) hydrogen-bond pattern (view direction [0 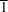 0]) in the crystal structure of leucopterin hemihydrate.

The chains run in the [120] and [ 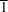 20] directions. The angle between neighbouring chains is 79.81° (see Fig. 13 ▸). The chains are stacked on top of each other with an interplanar distance of only 3.07 Å. In contrast, common interplanar distances between aromatic molecules are typically around 3.3–3.4 Å. The extremely small value found for leucopterin indicates that the packing is extremely dense. For further discussion, see Section 3.11.

Chains are connected by water molecules, which are tetrahedrally bonded to four different leucopterin molecules, *via* O—H⋯O and O⋯H—N hydrogen bonds. All possible hydrogen-bond donors and acceptors are involved in the hydrogen bonds. The water molecules are arranged in channels between the leucopterin molecules, like in a channel hydrate. However, the distances between the O atoms of the water molecules are 4.79 Å, so that there are no hydrogen bonds between the water molecules along the channel.

### 3.10. Crystal structure of leucopterin 0.2-hydrate

**Figure 14:**
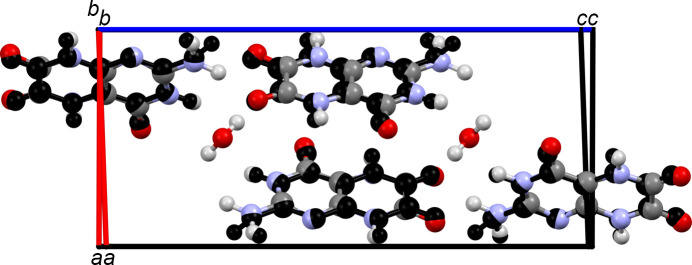
Overlay of the 0.2-hydrate (in black) and hemihydrate structures of leucopterin.

The crystal structure of the 0.2-hydrate is very similar to that of the hemihydrate. The lattice parameters differ only slightly (see Table 1 ▸) and also the general packing of leucopterin molecules is kept (see Fig. 14 ▸).

In the 0.2-hydrate, the hydrogen-bond pattern within the leucopterin chains and also the chain stacking are maintained. Neighbouring chains are connected only by N—H⋯O hydrogen bonds (see Fig. S9 in the supporting information).

Despite the removal of the water molecules, the unit-cell volumes determined by Rietveld refinement and DFT-D optimization decreased by only 12.47 and 4.84 Å^3^, respectively. These values are much smaller than the average space required by two water molecules, which is 48 Å^3^ (Glasser, 2020 ▸). Apparently, the molecular packing in the dehydrated form is less effective than in the hemihydrate. The channels which contain water molecules in the hemihydrate still exist in the dehydrated form. They do not collapse because of the strong interactions between the surrounding molecules, which prevent the molecules from moving into the void space.

### 3.11. Extremely high density

Leucopterin has an extremely high density. Under ambient conditions, the X-ray density of the hemihydrate is 1.9091 (4) kg dm^−3^; that of the 0.2-hydrate is about 1.89 kg dm^−3^ at room tem­per­ature. The density of the powder was investigated experimentally by suspending the powder in iodo­benzene. Despite the high density of iodo­benzene (ρ = 1.83 kg dm^−3^), the leucopterin particles did not move to the surface and the suspension did not separate, which agrees with the X-ray density. According to the rule of Kempster & Lipson (1972 ▸), non-H atoms have an average volume of 18 Å^3^ in organic crystals. For leucopterin, this value drops to ∼13 Å^3^. A more accurate estimation of average atomic volumes was performed by Hofmann (2002 ▸). Using these values, the molecular volume of leucopterin is estimated to be 201.79 Å^3^. The alternative approach of Mighell *et al.* (1987 ▸) yields a value of 195.64 Å^3^. In contrast, the experimental molecular volume of leucopterin is only 177.57 Å^3^. Many organic compounds have a density around 1 kg dm^−3^, *e.g.* hydro­carbons have a density around 0.7–1.1 kg dm^−3^ (*n*-decane = 0.73 kg dm^−3^ and naphthalene = 1.03 kg dm^−3^). Only a few organic compounds, consisting of C, N, H and O only, have a density of 1.909 kg dm^−3^ or higher under ambient conditions. Most of them are nitro compounds, which are frequently used as explosives (the explosive power is proportional to the fourth power of their density; that is why crystal engineering is applied to find new compounds and polymorphs of nitro compounds with the highest possible density). Non-nitro compounds with a density greater than 1.909 kg dm^−3^ under ambient conditions are rare. Examples include alloxan [Cambridge Structural Database (CSD; Groom *et al.*, 2016 ▸) refcode ALOXAN; Bolton, 1964 ▸], oxalic acid (OXALAC03; Derissen & Smith, 1974 ▸), croconic acid (GUMMUW; Braga *et al.*, 2002 ▸), octa­hydroxy­cyclo­butane (HOCBUT; Bock, 1968 ▸) and dodeca­hydroxy­cyclo­hexane dihydrate (SANTEH and SANTEH01; Klapötke *et al.*, 2005 ▸; Lim *et al.*, 2011 ▸). (The molecular formulae of all compounds are given in Fig. S10 in the supporting information.)

It is astonishing that even the dehydrated form of leucopterin has an extremely high density although the structure contains small voids at the position of the removed water molecule. According to the laws of Kitajgorodskij (1970 ▸), the space group *P*2/*c* does not allow for a dense packing due to the existence of the twofold axes. The rules of Kitajgorodskij only apply if the twofold axes are between the molecules. In the case of the hemihydrate, the water molecule is situated on the twofold axis, allowing a dense molecular packing. In contrast, in the anhydrate, there is no water molecule and the twofold axis is indeed between the molecules; the packing is thus not completely efficient. Nevertheless, the anhydrate has a very high density.

Why has leucopterin such a high density? There are several reasons:

– It contains a high number of N and O atoms, whereas CH, CH_2_ and CH_3_ groups are absent. N and O atoms are smaller and heavier than C atoms; the van der Waals diameter of N and O atoms is about 0.3–0.4 Å smaller than that of C atoms (Bondi, 1964 ▸; Chernyshov *et al.*, 2020 ▸); H and C atoms increase the relative volume. For example, caffeine (CSD refcode NIWFEE03; Fig. S10 in the supporting information), which has a similar molecular structure to leucopterin but contains three CH_3_ groups, has a density of only 1.448 kg dm^−3^ (Lehmann & Stowasser, 2007 ▸), probably due to the lower mass and weaker interactions of the CH_3_ groups.

– The high number of hydrogen bonds results in short intermolecular distances because N and O atoms exhibit even closer intermolecular distances if they are connected by hydrogen bonds.

These two arguments explain the high density of the compounds shown in Fig. 2 ▸. Furthermore, leucopterin chains are very efficiently stacked on top of each other, so that the molecules in one chain are positioned in the gap between the molecules in the neighbouring chains [Fig. S12(*a*) in the supporting information]. Additionally, atoms with partial positive charge (C and H) are positioned over atoms with partial negative charge (O and N) resulting in strong Coul­ombic interactions within close distances [Fig. S11(*b*) in the supporting information].

The high density and the pronounced intermolecular interactions lead to a high lattice energy, which explains the low solubility of leucopterin in water and organic solvents. The high density may explain the high refractive index of the beads containing leucopterin in the wing scales of *Pieris* butterflies.

## 4. Conclusions

The butterfly pigment leucopterin is a small and rigid molecule. However, the determination of its crystal structure and tautomeric state needed an enormous experimental and analytical effort, involving about 20 different methods: various syntheses and recrystallization attempts, DTA-TG, PXRD of various samples under ambient conditions, tem­per­ature-de­pen­dent PXRD, synchrotron powder diffraction (to obtain a reliable PDF), attempts to solve the crystal structure from powder data by indexing and direct-space methods, by local and global fit with deviating lattice parameters, and by global PDF fits, SCXRD, various SSNMR methods, including ^1^H MAS, ^13^C and ^15^N CPMAS, 2D ^1^H DQ MAS, lattice-energy optimizations with two different DFT-D methods on 17 possible tautomeric forms for assessing the lattice energies, the distortion of the crystal structure and the calculation of the ^1^H, ^13^C and ^15^N chemical shifts in the solid state. Most methods provided valuable information on the crystal structure and/or the tautomeric state. In other cases, the method itself worked well, but gave no or only limited insights. Actually, even more methods were applied than described in this article, *e.g.* IR spectroscopy, solution NMR and chromatographic methods.

The four main findings of these investigations are: (i) leucopterin is a variable hydrate, with a continuous transition from hemihydrate to (almost) anhydrate; (ii) it crystallizes in quite a rare space group (*P*2/*c*); (iii) in the solid state, it adopts the 2-amino-3,5,8-H tautomeric form; (iv) the molecules form an extremely efficient molecular packing resulting in an extraordinarily high density of 1.909 kg dm^−3^, which might explain the observed light scattering and opacity of the wings of *Pieris brassicae* and several other butterflies.

## Supplementary Material

Crystal structure: contains datablock(s) Leuco, global, Leuco_hydrate. DOI: 10.1107/S2052252523004281/lt5058sup1.cif

Structure factors: contains datablock(s) Leuco. DOI: 10.1107/S2052252523004281/lt5058Leucosup2.hkl

Rietveld powder data: contains datablock(s) Leuco_hydrate. DOI: 10.1107/S2052252523004281/lt5058Leuco_hydratesup3.rtv

Additional figures and tables. DOI: 10.1107/S2052252523004281/lt5058sup4.pdf

CCDC references: 2191321, 2191333

## Figures and Tables

**Table 1:** Crystallographic data of leucopterin hemihydrate and 0.2-hydrate

	**Hemihydrate**	**0.2-Hydrate**
Structure from	Single-crystal data	Powder data/Rietveld refinement
**Crystal data**		
Chemical formula	C_6_H_5_N_5_O_3_·0.5H_2_O	C_6_H_5_N_5_O_3_·∼0.2H_2_O^*a*^
CCDC reference	2191333	2191321
*M*_r_	204.16	198.5 (33)^*b*^
Crystal system	monoclinic	monoclinic
Space group (No.)	*P*2/*c* (13)	*P*2/*c* (13)
*Z*, *Z’*	4, 1	4, 1
Temperature (K)	296 (2)	296 (2)
*a* (Å)	8.0781 (10)	8.1026 (4)
*b* (Å)	4.7930 (6)	4.8366 (3)
*c* (Å)	18.345 (2)	17.8289 (14)
α (°)	90.	90.
β (°)	90.223 (8)	88.531 (4)
γ (°)	90.	90.
*V* (Å^3^)	710.28 (15)	698.46 (8)
ρ_calc_ (kg dm^−3^)	1.9091 (4)	1.888 (32)^*b*^
Radiation type	Cu *K*α	Cu *K*α_1_
Wavelength (Å)	1.54178	1.5406
μ (mm^−1^)	1.392	1.365
		
**Data collection**		
Crystal size (mm)	0.03 × 0.03 × 0.002	powder
Diffractometer	Siemens/Bruker three-circle	Stoe Stadi-P
Specimen mounting	glass pin	0.7 mm capillary
Data collection mode	CCD detector	step
Detector	APEXII CCD	linear PSD
2θ_min_ (°)	–	4
2θ_max_ (°)	–	70
2θ_step_ (°)	–	0.2
		
**Refinement**		
*R*1	14.6%	–
*wR*2	44.5%	–
*R*_p_ (%)	–	2.516
*R*_wp_ (%)	–	3.454
*R*_exp_ (%)	–	2.171
*R*_p_′ (%)^*c*^	–	10.091
*R*_wp_′ (%)^*c*^	–	10.517
*R*_exp_′ (%)^*c*^	–	6.609
GOF	2.41	1.591
No. of data points	–	7800
No. of reflections in refinement	309	310
No. of refined parameters	57	101
No. of restraints	2	52
H-atom treatment	calculated	refined with restraints

Notes: (*a*) The water content is uncertain; it could also be 0.1 or 0. (*b*) The high uncertainties of these values are caused by the uncertain amount of water in the structure. (*c*) The *R*_p_′, *R*_wp_′ and *R*_exp_′ values are background-corrected data according to Coelho (2004 ▸).

**Table 2:** Comparison of the experimental and computed crystallographic data of hemihydrate, 0.2-hydrate and anhydrate leucopterin

	Hemihydrate			0.2-Hydrate	Anhydrate	
Structure from	Single-crystal data	DFT-D opt. B86r	DFT-D opt. B88	Powder data/Rietveld refinement	DFT-D opt. B86r	DFT-D opt. B88
Chemical formula	C_6_H_5_N_5_O_3_·0.5H_2_O	C_6_H_5_N_5_O_3_·0.5H_2_O	C_6_H_5_N_5_O_3_·0.5H_2_O	C_6_H_5_N_5_O_3_·∼0.2H_2_O	C_6_H_5_N_5_O_3_	C_6_H_5_N_5_O_3_
CCDC reference	2191333	–	–	2191321	–	–
*M*_r_	204.16	204.16	204.16	198.5	195.14	195.14
Crystal system	monoclinic	monoclinic	monoclinic	monoclinic	monoclinic	monoclinic
Space group (No.)	*P*2/*c* (13)	*P*2/*c* (13)	*P*2/*c* (13)	*P*2/*c* (13)	*P*2/*c* (13)	*P*2/*c* (13)
*Z*, *Z*′	4, 1	4, 1	4, 1	4, 1	4, 1	4, 1
Temperature (K)	296 (2)	0	0	296 (2)	0	0
*a* (Å)	8.0781 (10)	7.9454	8.0910	8.1026 (4)	7.9446	8.1303
*b* (Å)	4.7930 (6)	4.7492	4.7638	4.8366 (3)	4.8086	4.8111
*c* (Å)	18.345 (2)	18.3465	18.4401	17.8289 (14)	18.0034	18.1146
α (°)	90	90	90	90	90	90
β (°)	90.223 (8)	90.5280	90.5185	88.531 (4)	88.4353	88.5662
γ (°)	90	90	90	90	90	90
*V* (Å^3^)	710.28 (15)	692.263	710.724	698.46 (8)	687.517	708.339
ρ_calc_ (kg dm^−3^)	1.9091 (4)	1.95874	1.90784	1.888 (32)	1.88522	1.82980

**Table 3:** ^1^H, ^13^C and ^15^N computed and experimental chemical shifts, peak assignments and RMSE values of leucopterin hemihydrate and anhydrate containing tautomer T1 ‘Calc’ refers to the chemical shifts computed with the optB88 method. See Scheme 1 for atom numbering.

	Hemihydrate						Anhydrate					
	^1^H (ppm)		^13^C (ppm)		^15^N (ppm)		^1^H (ppm)		^13^C (ppm)		^15^N (ppm)	
#	Exp	Calc	Exp	Calc	Exp	Calc	Exp	Calc	Exp	Calc	Exp	Calc
1					154.9	155.3					154.9	155.0
2			153.4	152.0					154.1	152.2		
3	10.2	10.0			137.5	133.5	7.9	7.6			146.8	149.4
4			156.2	154.4					156.0	153.5		
4a			99.3	102.6					99.6	103.8		
5	10.2	11.1			126.8	131.4	9.8	11.1			135.9	131.5
6			153.4	152.2					154.1	152.2		
7			157.6	157.9					157.8	158.4		
8	11.6	12.4			146.9	149.8	11.8	13.2			124.4	130.5
8a			142.4	143.4					142.3	143.7		
9	6.9, 7.9	6.9, 7.5			81.0	77.1	6.8, 7.9	7.4, 7.6			79.9	75.5
H_2_O	3.4	2.5										

**Table 4:** Experimental (SCXRD) and computed unit-cell parameters (*P*2/*c*, *Z* = 4) for the structural models of leucopterine (T1 and T8) hemihydrate Relative energies with respect to T1. ‘Gas’: single molecule in the gas phase, by *GAUSSIAN09*; ‘Solid’: in the solid state, by *Quantum ESPRESSO* with the two vdW-DF2 methods optB88. ^1^H, ^13^C and ^15^N chemical shift RMSEs for the computed structures.

Structure	Δ*E* (kJ mol^−1^)		^1^H RMSE (ppm)	^13^C RMSE (ppm)	^15^N RMSE (ppm)	Volume (Å^3^)	*a* (Å)	*b* (Å)	*c* (Å)	β (°)
	Gas	Solid								
SCXRD	–	–	–	–	–	710.274	8.0781	4.7930	18.3452	90.2238
T1	0.00	0.00	0.6	1.8	3.5	710.724	8.091	4.764	18.440	90.519
T8	18.28	126.64	0.6	5.2	27.6	859.248	7.796	6.385	18.534	68.644

## Footnotes

1 Apart from *Pieris brassicae* and *Pieris napi*, leucopterin is also found in other species of Pieridae, *e.g.* in *Pieris rapae* (small white or small cabbage white, in German ‘Kleiner Kohlweißling’), *Anthocharis cardamines* (orange tip, in German ‘Aurorafalter’) and *Gonepteryx rhamni* (common brimstone, in German ‘Zitronenfalter’). Additionally, leucopterin is found in wasps, *e.g. Vespa crabro* (European Hornet, in German ‘Hornisse’), *Vespula germanica* (German wasp, in German ‘Deutsche Wespe’) and *Vespula vulgaris* (common wasp, in German ‘Gemeine Wespe’) (Schöpf & Becker, 1936 ▸).
