# Supplementary material for: Leucopterin, the white pigment in butterfly wings: structural analysis by PDF fit, FIDEL fit, Rietveld refinement, solid-state NMR and DFT-D
Source: IUCrJ. 2023 Jun 17;10(Pt 4):448–63. doi: 10.1107/S2052252523004281 (PMC10324491; doi:10.1107/S2052252523004281)
Supplement: Supplementary file 4 [file m-10-00448-sup4.pdf]

IUCrJ

Volume 10 (2023)

Supporting information for article:

Leucopterin, the white pigment in butterfly wings: structural analysis by PDF fit, FIDEL fit, Rietveld refinement, solid-state NMR and DFT-D

Federica Bravetti, Lukas Tapmeyer, Kathrin Skorodumov, Edith Alig, Stefan Habermehl, Robert Hühn, Simone Bordignon, Angelo Gallo, Carlo Nervi, Michele R. Chierotti and Martin U. Schmidt

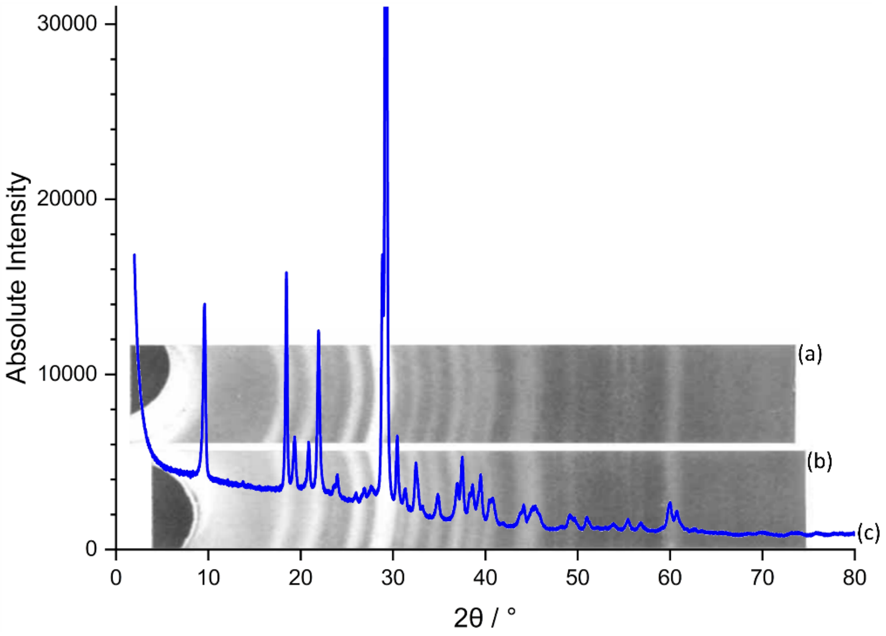

Figure S1 Comparison of the Debye-Scherrer patterns of (a) synthetic leucopterin from Purrmann (1940), (b) natural leucopterin isolated from butterflies, and (c) our synthetic leucopterin (blue curve). The images of the Debye-Scherrer films in (a) and (b) were taken from Purrmann (1940).

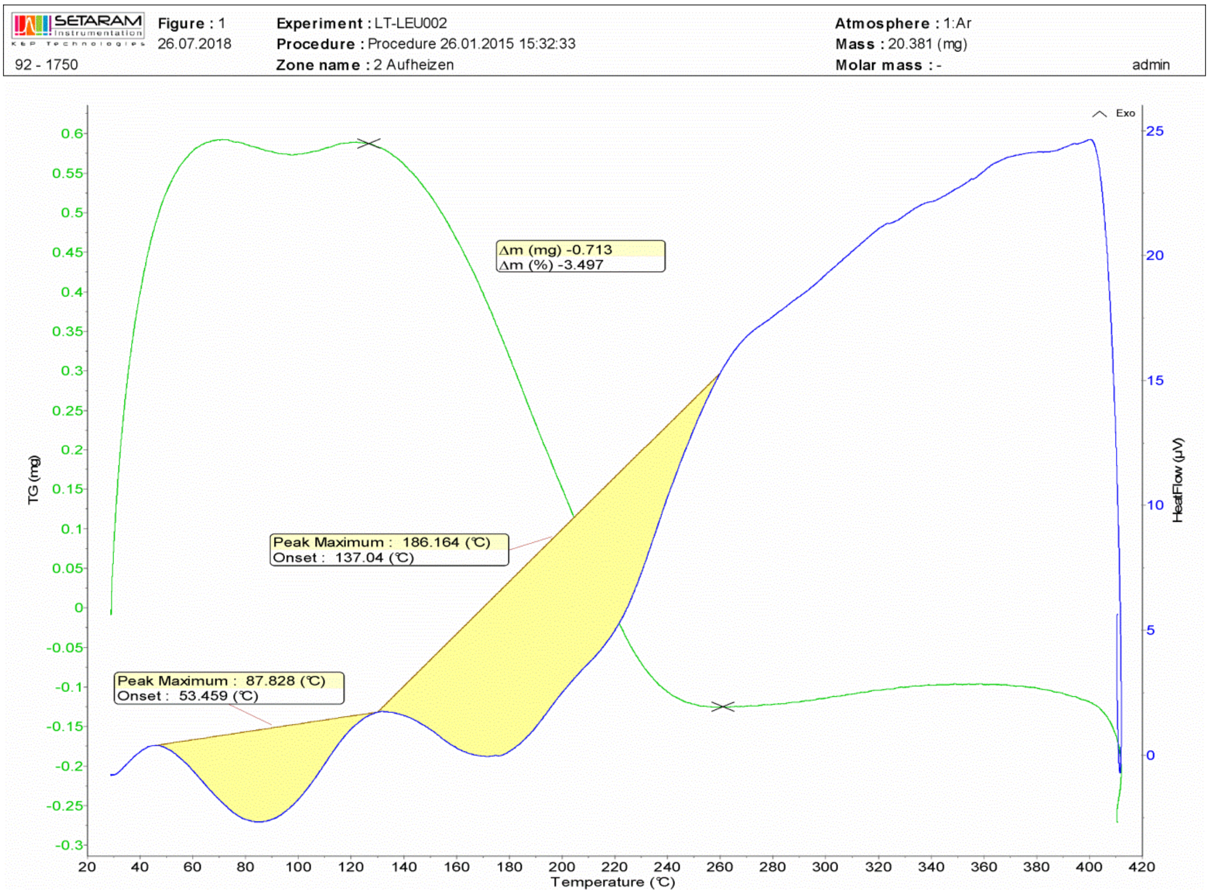

Figure S2 DTA-TG of leucopterin hemihydrate.

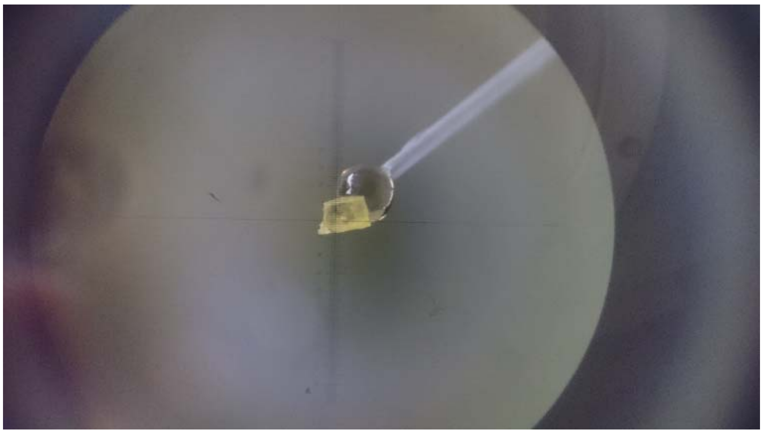

Figure S3 Single crystal of leucopterin hemihydrate on a glass pin.

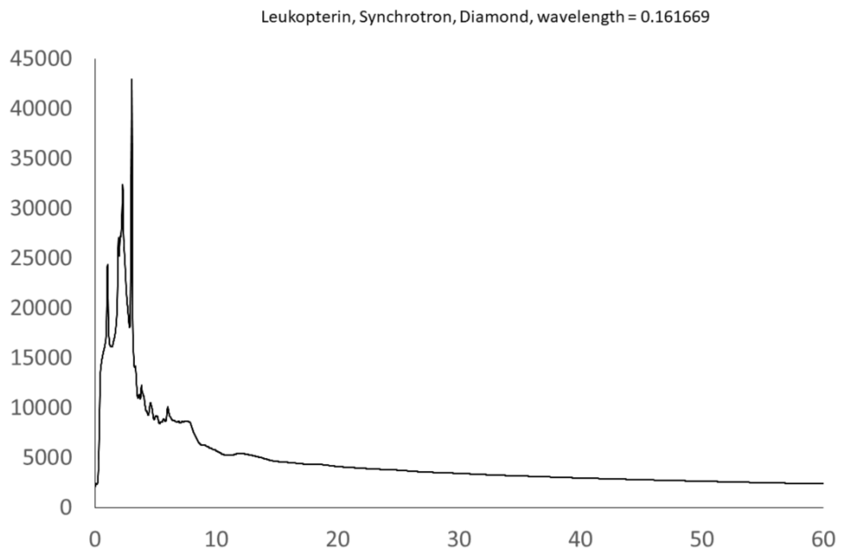

Figure S4 Synchrotron powder diffraction data.

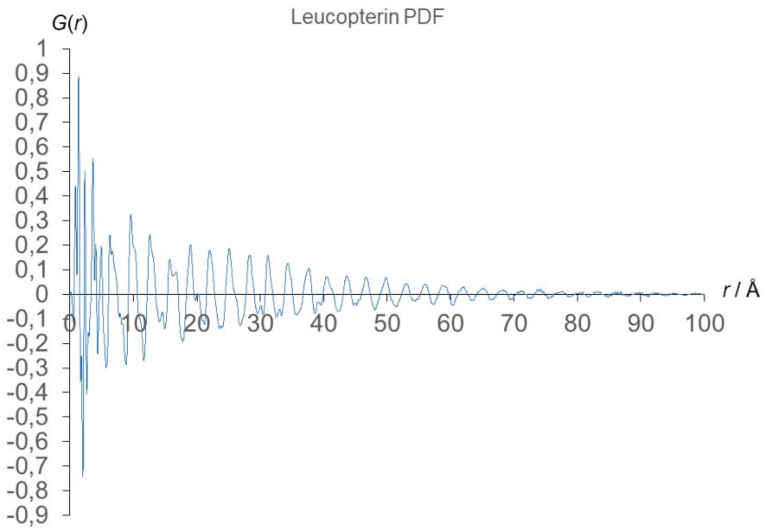

Figure S5 Experimental PDF.

PDF global fits

PDF global fits were performed with the aim to solve the crystal structure of the hemihydrate. All PDF fits were performed without the water molecule. At first, PDF fits were run in various space groups, not including *P*2/*c*. The best resulting fit is shown in Figure S6a. The corresponding crystal structure was wrong. After *P*2/*c* turned out to be the correct space group, additional PDF global fits were run in *P*2/*c*. The R_{wp}^{PDF} value dropped, and the best fit (Figure 4 in the main text) corresponds to the correct structure. Figure S6b gives an overlay of the structures from PDF fit and single-crystal data.

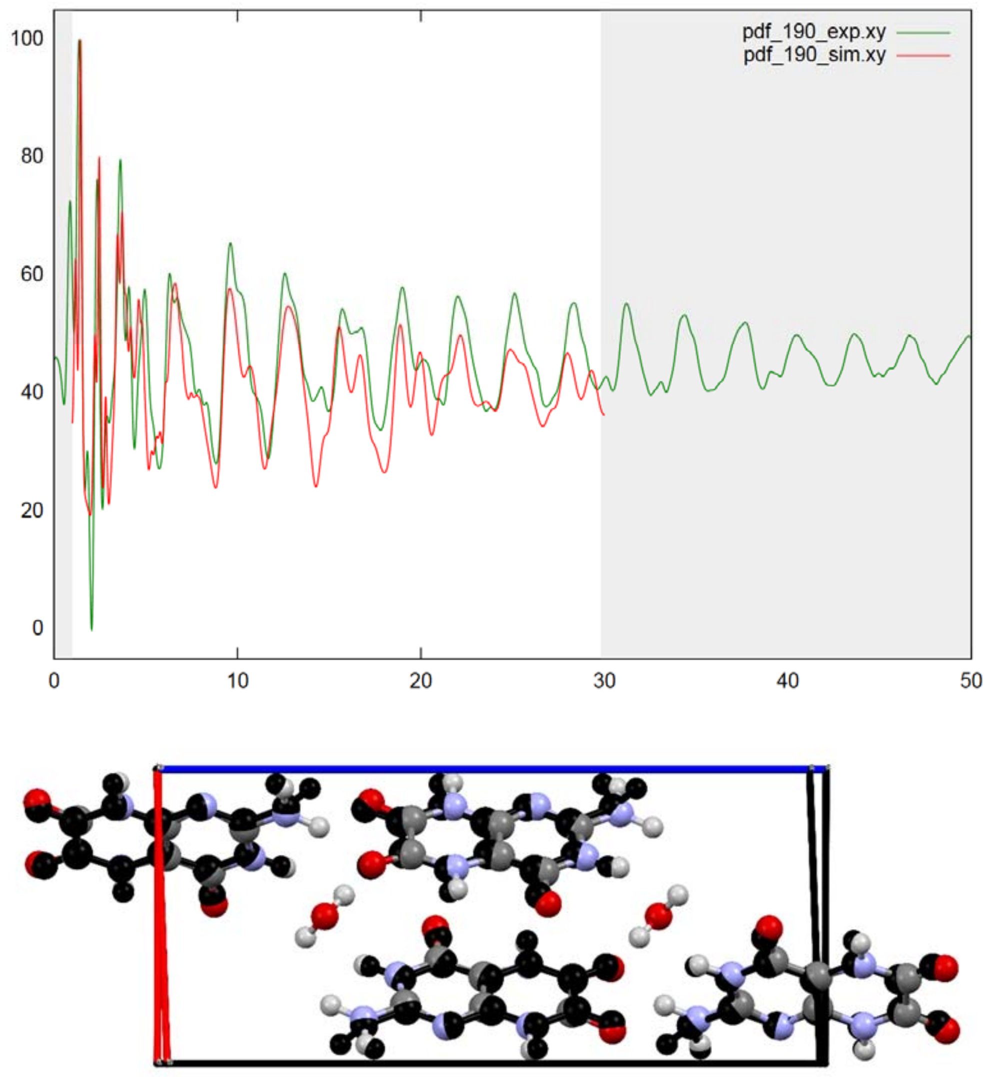

Figure S6 PDF global fit for structure solution of the hemihydrate. (a) Best PDF fit obtained in other space groups except *P*2/*c*: space group P2$_1$/c, *a* = 4.82 Å , *b* = 3.92 Å, c = 41.22 Å, *β* = 108.4°, *V* = 738.82 Å^3. This crystal structure is wrong. (b) Structure from the best PDF fit obtained in *P*2/*c*: *a* = 8.08 Å, *b* = 4.82 Å, c = 17.94 Å, *β* = 88.0°, *V* = 700.60 Å^3. R_{wp}^{PDF} = 38.05 %. The structure from PDF fit is drawn in black, and overlayed with the structure from single-crystal data (in colour).

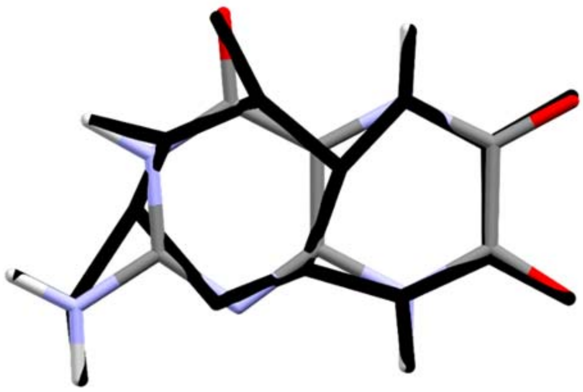

Figure S7 Overlay of the distorted molecule of leucopterin 0.2-hydrate after the unrestrained refinement (in black) with the correct one (coloured). During the free refinement, the occupation of the oxygen atom representing the water molecule dropped from 0.42 to 0.212(11), corresponding to a 0.08-hydrate.

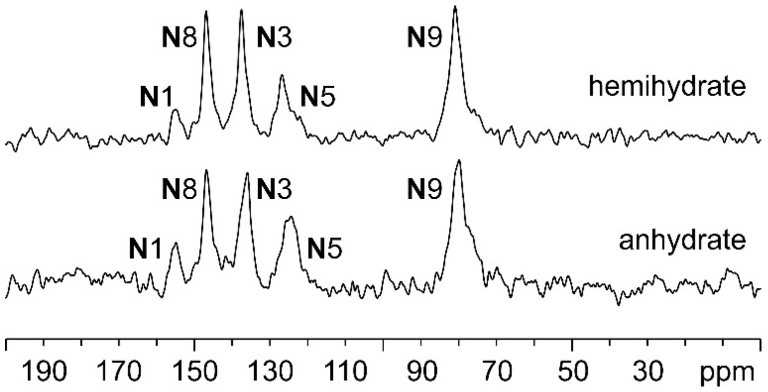

Figure S8 Assigned ^{15}N CPMAS spectra (contact time 4 ms) of leucopterin hemihydrate and anhydrate.

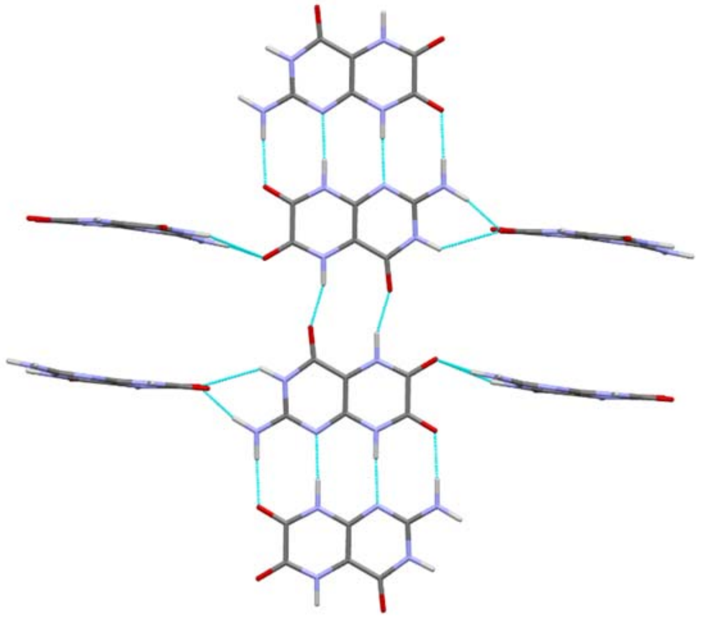

Figure S9 Molecular chains in the crystal structure of leucopterin anhydrate, after DFT-D optimisation with fixed lattice parameters. Colour code in all drawings: C = grey, O = red, N = blue, H = white, hydrogen bonds = turquoise. View direction [120].

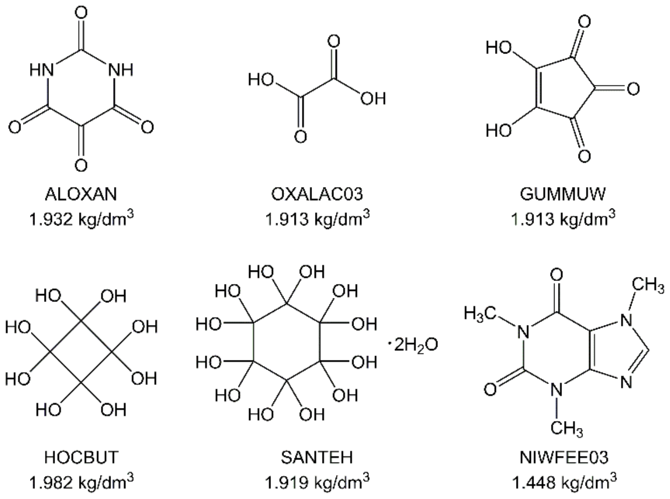

Figure S10 Molecular structures, CCDC refcodes and densities of the crystal structures of some non-nitro compounds with a density higher than 1.909 kg/dm^3 at ambient conditions, and caffeine.

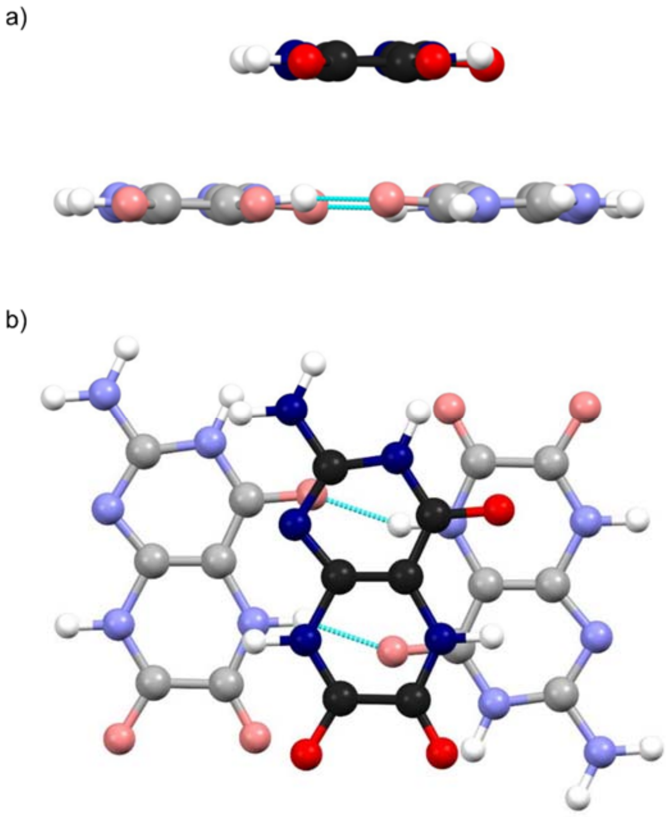

Figure S11 Molecular packing in leucopterin hemihydrate. (a) Stacking of molecules in neighbouring chains. (b) Perpendicular view. One molecule is highlighted.

Table S1 ^{1}H, ^{13}C and ^{15}N computed and experimental chemical shifts, peak assignments and RMSE values of leucopterin hemihydrate containing tautomer T1. Calc 1 and Calc 2 refer to the chemical shifts computed with the B86r or optB88 method, respectively. See Scheme 1 for atom numbering (#).

	**^{1}H chemical shift (ppm)**			**^{13}C chemical shift (ppm)**			**^{15}N chemical shift (ppm)**		
**#**	**Exp**	**Calc 1**	**Calc 2**	**Exp**	**Calc 1**	**Calc 2**	**Exp**	**Calc 1**	**Calc 2**
1							154.9	152.4	155.3
2				153.4	151.6	152.0			
3	10.2	9.9	10.0				137.5	133.0	133.5
4				156.2	154.5	154.4			
4a				99.3	102.6	102.6			
5	10.2	11.2	11.1				126.8	131.9	131.4
6				153.4	152.1	152.2			

7				157.6	158.3	157.9			
8	11.6	12.8	12.4				146.9	150.1	149.8
8a				142.4	143.2	143.4			
9	6.9<br>7.9	6.9<br>7.4	6.9<br>7.5				81.0	79.7	77.1
H_2O	3.4	2.3	2.5						

Table S2 ^{1}H, ^{13}C and ^{15}N computed and experimental chemical shifts, peak assignments and RMSE values of leucopterin anydrate containing tautomer T1. Calc 1 and Calc 2 refer to the chemical shifts computed with the B86r or optB88 method, respectively. See Scheme 1 for atom numbering (#).

	^{1}H chemical shift (ppm)			^{13}C chemical shift (ppm)			^{15}N chemical shift (ppm)		
**#**	**Exp**	**Calc 1**	**Calc 2**	**Exp**	**Calc 1**	**Calc 2**	**Exp**	**Calc 1**	**Calc 2**
1							154.9	152.4	155.0
2				154.1	152.0	152.2			
3	7.9	7.5	7.6				146.8	149.7	149.4
4				156.0	153.7	153.5			
4a				99.6	103.7	103.8			
5	9.8	11.3	11.1				135.9	131.0	131.5
6				154.1	152.1	152.2			
7				157.8	158.7	158.4			
8	11.8	13.6	13.2				124.4	130.4	130.5
8a				142.3	143.6	143.7			
9	6.8<br>7.9	7.2<br>7.2	7.4<br>7.6				79.9	78.3	75.5

Table S3 Experimental (SCXRD) and computed cell parameters (*P*2/*c*, *Z* = 4) for the 17 structural models of leucopterine (T1-T17) hemihydrate, each one containing a different tautomer. Relative energies with respect to T1. Gas: single molecule in the gas phase, by Gaussian 09; Solid: in the solid state, by Quantum Espresso with the two vdW-DF2 methods B86r (in black) and optB88 (in red). ^{1}H, ^{13}C and ^{15}N chemical shift RMSEs for the computed structures.

**Structure**	**ΔE (kJ/mol)**		**^{1}H RMSE (ppm)**	**^{13}C RMSE (ppm)**	**^{15}N RMSE (ppm)**	**Volume (Å³)**	***a* (Å)**	***b* (Å)**	***c* (Å)**	***β* (°)**
	**Gas**	**Solid**								
SCXRD	/	/	/	/	/	710.274	8.0781	4.7930	18.3452	90.2238
T1	0.00	0.00<br>0.00	0.8<br>0.6	1.8<br>1.8	3.6<br>3.5	692.263<br>710.724	7.945<br>8.091	4.749<br>4.764	18.346<br>18.440	90.528<br>90.519
T2	2.24	40.11*<br>62.29	1.3<br>2.8	1.7<br>1.6	4.6<br>10.2	763.008<br>780.738	10.893<br>10.609	4.757<br>4.922	17.803<br>18.303	55.908<br>54.769
T9	17.25	84.21<br>86.15	0.8<br>0.7	5.8<br>6.0	35.1<br>36.9	683.122<br>703.755	7.423<br>7.546	5.067<br>5.106	18.192<br>18.296	86.695<br>86.575

T7	14.75	88.19<br>85.04	1.4<br>1.2	2.6<br>2.6	13.1<br>12.6	738.629<br>759.854	9.304<br>9.433	4.717<br>4.727	17.954<br>18.036	110.379<br>109.125
T11	9.04	89.79<br>87.43	1.5<br>1.5	6.8<br>6.8	48.1<br>49.7	727.800<br>750.855	8.442<br>8.625	4.804<br>4.815	17.953<br>18.088	91.777<br>91.702
T4	11.20	90.72<br>86.90	1.2<br>1.2	5.4<br>5.4	44.8<br>46.3	738.546<br>763.247	8.263<br>8.519	4.857<br>4.836	18.418<br>18.544	92.214<br>92.385
T17	32.16	96.18<br>97.57	1.1<br>1.4	4.4<br>4.3	37.4<br>39.0	717.726<br>746.747	10.460<br>10.162	3.722<br>3.950	18.901<br>19.012	102.765<br>101.914
T15	9.41	98.42<br>99.34	1.6<br>1.5	2.3<br>2.4	25.3<br>27.2	712.686<br>738.174	8.005<br>8.233	4.690<br>4.683	18.993<br>19.150	91.683<br>91.229
T14	21.43	122.66<br>121.62	0.4<br>0.4	10.9<br>10.9	60.0<br>52.7	725.651<br>751.741	7.474<br>7.578	5.291<br>5.374	18.441<br>18.576	95.751<br>96.444
T8	18.28	126.94<br>126.64	1.8<br>0.6	4.9<br>5.2	24.8<br>27.6	803.216<br>859.248	8.550<br>7.796	6.011<br>6.385	17.591<br>18.534	62.801<br>68.644
T6	16.43	127.18<br>123.70	0.7<br>0.7	5.8<br>5.8	18.9<br>20.4	736.255<br>758.263	8.888<br>9.056	4.416<br>4.427	18.772<br>18.923	92.195<br>91.920
T3	15.28	137.38<br>136.86	3.1<br>2.1	2.6<br>2.1	33.0<br>36.1	738.481<br>770.354	9.406<br>9.533	5.479<br>5.563	16.643<br>16.788	59.432<br>59.917
T13	18.02	143.06<br>136.43	0.6<br>0.7	8.4<br>8.2	50.1<br>51.4	825.023<br>860.129	8.119<br>8.330	5.182<br>5.222	19.881<br>20.114	99.503<br>100.556
T5	23.00	151.01<br>116.25	1.3<br>1.8	4.2<br>4.9	27.8<br>37.3	761.000<br>824.300	8.467<br>10.835	5.167<br>4.483	17.825<br>18.395	102.654<br>112.687
T12	26.36	155.06<br>154.76	1.1<br>1.1	10.1<br>10.01	62.7<br>64.1	716.744<br>742.323	7.491<br>7.595	5.324<br>5.404	17.980<br>18.097	88.269<br>88.083
T16	20.79	165.18<br>164.44	1.3<br>1.2	4.3<br>4.3	39.1<br>41.1	729.309<br>758.472	9.377<br>9.629	4.555<br>4.608	17.179<br>17.220	96.360<br>96.888
T10	30.68	206.76<br>200.30	0.8<br>0.9	9.4<br>9.4	54.4<br>56.5	835.577<br>877.585	7.084<br>7.213	6.604<br>6.792	18.146<br>18.259	100.193<br>101.173

*During the DFT-D optimisation process, the T2 tautomer was converted into T1, but the molecular arrangement is different. This process proceeded via a rearrangement of the molecules in the cell (there is a significant change in the β angle of the optimised cell) which however resulted in a higher energy. This rearrangement allowed for the transformation of the O–H···N intramolecular interaction of T2 into the O···H–N intramolecular interaction of T1. The same interaction is present in T1, but the optimised T2 cell is very different from the T1 one. The T1 optimised cell parameters fit very well with the experimental data.
